# Hepatocyte TMEM16A Deletion Retards NAFLD Progression by Ameliorating Hepatic Glucose Metabolic Disorder

**DOI:** 10.1002/advs.201903657

**Published:** 2020-03-20

**Authors:** Jia‐Wei Guo, Xiu Liu, Ting‐Ting Zhang, Xiao‐Chun Lin, Yu Hong, Jie Yu, Qin‐Yan Wu, Fei‐Ran Zhang, Qian‐Qian Wu, Jin‐Yan Shang, Xiao‐Fei Lv, Jing‐Song Ou, Jia‐Guo Zhou, Rui‐Ping Pang, Bao‐Dong Tang, Si‐Jia Liang

**Affiliations:** ^1^ Department of Pharmacology Cardiac and Cerebral Vascular Research Center Zhongshan School of Medicine Sun Yat‐Sen University Guangzhou 510080 China; ^2^ Department of Gastrointestinal Surgery The First Affiliated Hospital Sun Yat‐Sen University Guangzhou 510080 China; ^3^ Department of Gastroenterology The First People's Hospital of Foshan Foshan 528000 China; ^4^ Key Laboratory of Metabolic Cardiovascular Diseases Research of National Health Commission Ningxia Medical University Yinchuan 750004 China; ^5^ Division of Cardiac Surgery The Key Laboratory of Assisted Circulation Ministry of Health The First Affiliated Hospital Sun Yat‐Sen University Guangzhou 510080 China; ^6^ National‐Guangdong Joint Engineering Laboratory for Diagnosis and Treatment of Vascular Diseases The First Affiliated Hospital Sun Yat‐Sen University Guangzhou 510080 China; ^7^ Program of Kidney and Cardiovascular Disease The Fifth Affiliated Hospital Sun Yat‐Sen University Guangzhou 510080 China; ^8^ Department of Cardiology Sun Yat‐Sen Memorial Hospital Sun Yat‐Sen University Guangzhou 510120 China; ^9^ Guangdong Province Key Laboratory of Brain Function and Disease Zhongshan School of Medicine Sun Yat‐Sen University Guangzhou 510080 China; ^10^ Department of Physiology Pain Research Center Zhongshan School of Medicine Sun Yat‐Sen University Guangzhou 510080 China; ^11^ Department of Gastroenterology The First Affiliated Hospital Sun Yat‐Sen University Guangzhou 510080 China

**Keywords:** glucose metabolic disorder, GLUT2, hepatic steatosis, nonalcoholic fatty liver disease, TMEM16A, VAMP3

## Abstract

Nonalcoholic fatty liver disease (NAFLD) is the most prevalent form of chronic liver disease, and the mechanisms underpinning its pathogenesis have not been completely established. Transmembrane member 16A (TMEM16A), a component of the Ca^2+^‐activated chloride channel (CaCC), has recently been implicated in metabolic events. Herein, TMEM16A is shown to be responsible for CaCC activation in hepatocytes and is increased in liver tissues of mice and patients with NAFLD. Hepatocyte‐specific ablation of TMEM16A in mice ameliorates high‐fat diet‐induced obesity, hepatic glucose metabolic disorder, steatosis, insulin resistance, and inflammation. In contrast, hepatocyte‐specific TMEM16A transgenic mice exhibit the opposite phenotype. Mechanistically, hepatocyte TMEM16A interacts with vesicle‐associated membrane protein 3 (VAMP3) to induce its degradation, suppressing the formation of the VAMP3/syntaxin 4 and VAMP3/synaptosome‐associated protein 23 complexes. This leads to the impairment of hepatic glucose transporter 2 (GLUT2) translocation and glucose uptake. Notably, VAMP3 overexpression restrains the functions of hepatocyte TMEM16A in blocking GLUT2 translocation and promoting lipid deposition, insulin resistance, and inflammation. In contrast, VAMP3 knockdown reverses the beneficial effects of TMEM16A downregulation. This study demonstrates a role for TMEM16A in NAFLD and suggests that inhibition of hepatic TMEM16A or disruption of TMEM16A/VAMP3 interaction may provide a new potential therapeutic strategy for NAFLD.

## Graphical abstract

Transmembrane member 16A (TMEM16A) is an essential component of the hepatocyte calcium‐activated chloride channel and is increased in livers with hepatic steatosis. It interacts with vesicle‐associated membrane protein 3 to disrupt soluble Nethylmaleimide‐sensitive factor attachment protein receptors formation, thereby limiting glucose transporter 2 translocation and hepatic glucose uptake. Liver‐specific deficiency of TMEM16A ameliorates glucose metabolic disorder and nonalcoholic fatty liver disease.

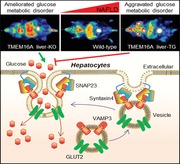

## 1 Introduction

Nonalcoholic fatty liver disease (NAFLD) encompasses simple steatosis, nonalcoholic steatohepatitis (NASH), fibrosis, cirrhosis, and hepatocellular carcinoma.^[^ ^1^, ^2^ ^]^ Hepatic lipid accumulation (steatosis) and insulin resistance reflect the early stages of NAFLD, which spontaneously triggers an inflammatory response.^[^ ^3^, ^4^, ^5^ ^]^ Insulin resistance is mediated by impaired insulin signaling, such as disruption of the insulin receptor substrate 1 (IRS1)/AKT pathway, and leads to insulin insensitivity and glucose intolerance.^[^ ^1^, ^4^ ^]^ Additionally, proinflammatory mediators (e.g., cytokines), together with macrophage infiltration in the liver, exert direct effects on hepatocytes to induce inflammation and exacerbate hepatic steatosis; this promotes NASH progression by increasing the expression of cholesterol synthesis‐related genes and fatty acid synthesis and by decreasing the expression of fatty acid oxidation‐related genes.^[^ ^1^, ^4^ ^]^ While these pathogenic processes are suggested to contribute to the establishment of steatohepatitis, the underlying mechanisms that occur during NAFLD progression remain largely obscure.

The liver is a key organ for maintaining glucose homeostasis, converting almost one third of glucose from the blood to glycogen after the ingestion of a meal.^[^ ^6^ ^]^ Glucose transporters (GLUTs) are a group of plasma membrane proteins that facilitate glucose transport across the cell membrane.^[^ ^7^, ^8^ ^]^ Among the GLUT subtypes, GLUT2 is abundantly expressed in specific tissues including the liver, intestine, and pancreas.^[^ ^7^, ^9^ ^]^ During the pathogenesis of NAFLD, disruption of insulin signaling results in hepatic steatosis and is accompanied by impairment of GLUT2 translocation to the hepatocyte plasma membrane.^[^ ^10^ ^]^ The subsequent impairment of GLUT2‐mediated glucose uptake and ensuing conversion to glycogen are suggested to be responsible for ectopic liver fat deposition. This is thought to occur via the conversion of excess carbohydrates to fatty acids through the de novo lipogenesis (DNL) pathway, which in turn further contributes to hepatic steatosis.^[^ ^6^, ^7^ ^]^ Therefore, GLUT2 expression and/or its translocation in hepatocytes play an important role in regulating glucose homeostasis and insulin sensitivity. Although the mechanisms of GLUT2 protein expression, subcellular trafficking, and translocation have been reported,^[^ ^9^, ^10^ ^]^ the regulation of GLUT2 in hepatocytes is not well understood.

An increasing number of studies from other groups and ours have demonstrated that chloride (Cl^−^) channels involve various pathological processes including lipid metabolic disorder, gut inflammation, and liver diseases.^[^ ^3^, ^11^, ^12^, ^13^, ^14^ ^]^ Blockade of chloride channels prevented the entrance of superoxide anion radicals into hepatic stellate cells (HSCs) and inhibited HSCs activation, which is critical to fibrosis development.^[^ ^13^ ^]^ Patients with cystic fibrosis have shown abnormal hepatic lipid metabolism due to dysfunction of the epithelial chloride channel cystic fibrosis transmembrane regulator (CFTR).^[^ ^14^ ^]^ Lubiprostone, an activator of type 2 chloride channel (ClC‐2) is suggested to be a potential treatment for NAFLD patients with constipation through the suppression of gut permeability, providing anti‐inflammatory benefits in liver.^[^ ^15^ ^]^ Meanwhile, specific knockdown of ClC‐2 in liver protected against high‐fat diet (HFD)‐induced hepatic steatosis.^[^ ^3^ ^]^ These findings indicate that Cl^−^ channels may be a critical regulator of liver function. Ca^2+^‐activated Cl^−^ channels (CaCC) are ubiquitously expressed and suggested to regulate a variety of physiological activities.^[^ ^16^, ^17^, ^18^ ^]^ Transmembrane member 16A (TMEM16A) is a member of the TMEM16 family and identified as an essential component of CaCC in many cell types.^[^ ^17^, ^18^, ^19^ ^]^ We previously demonstrated that TMEM16A plays an important role in regulating endothelial oxidative stress, smooth muscle cell proliferation, cerebrovascular remodeling, and progression of hypertension.^[^ ^17^, ^19^ ^]^ Additionally, selective renal knockout of TMEM16A was associated with a mild proteinuric phenotype.^[^ ^20^ ^]^ TMEM16A was also involved in mediating glucose‐stimulated insulin secretion in islet β cells,^[^ ^21^, ^22^ ^]^ indicating that TMEM16A may also play a role in metabolic syndromes. Although the existence of CaCC in hepatocytes has been demonstrated,^[^ ^23^ ^]^ the functional role of TMEM16A/CaCC in the liver is unknown. The present study shows that TMEM16A is the abundantly expressed Cl^−^ channel in livers, and its expression is increased in the fatty livers of mice and patients with hepatic steatosis. We further generated hepatocyte‐specific TMEM16A knockout or transgenic mice, and the results demonstrated a prosteatotic effect of TMEM16A in the pathogenesis of NAFLD.

## 2 Results

### 2.1 TMEM16A Expression Correlates with Hepatic Steatosis

**Figure 1:**
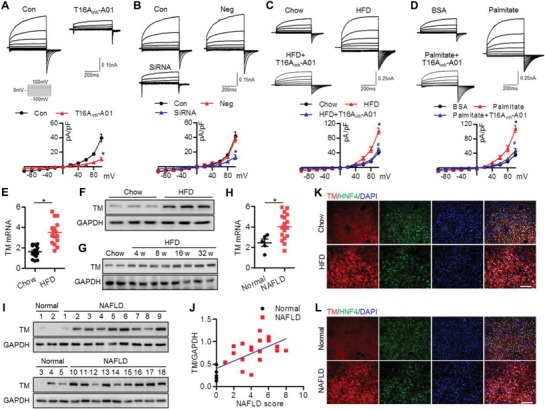
TMEM16A expression is increased in livers with hepatic steatosis. A,B) Representative traces of whole‐cell patch‐clamp recordings of *I*_Cl.Ca_ in primary hepatocytes treated with T16Ainh‐A01 (10 µmol L^−1^) (A) or in hepatocytes transfected with negative siRNA (Neg) or TMEM16A siRNA (40 nmol L^−1^) (B) for 24 h. *I*_Cl.Ca_ was recorded in the presence of 500 nmol L^−1^ [Ca^2+^]i. *I*–*V* curves of *I*_Cl.Ca_ are shown. *P < 0.05 versus 500 nmol L^−1^ [Ca^2+^]i (control), *n* = 6. C,D) *I*_Cl.Ca_ evoked by 500 nmol L^−1^ [Ca^2+^]i was potentiated in hepatocytes isolated from mice after feeding HFD for 32 weeks (C) or stimulated with palmitate (200 µmol L^−1^) for 24 h in vitro (D), which was inhibited by T16Ainh‐A01. *P < 0.05 versus chow or BSA, #*P* < 0.05 versus HFD or palmitate, *n* = 6. E) TMEM16A (TM) mRNA level in liver tissues of mice fed with chow diet or HFD for 32 weeks. *P < 0.05 versus chow, *n* = 16 per group. F) Western blotting of TMEM16A in livers from mice after chow diet or HFD treatment for 32 weeks, *n* = 6 per group. G) Representative western blotting of TMEM16A expression in livers of mice after HFD for the indicated time periods, *n* = 6 per group. H,I) TMEM16A mRNA (H) and protein (I) levels in livers from normal individuals (*n* = 5) or NAFLD patients (*n* = 18). *P < 0.05 versus normal. J) Pearson correlation analysis of the relationship between TMEM16A protein expression and NAFLD score in human subjects (*r* = 0.6824, *P* = 0.0003). K,L) Immunofluorescence of TMEM16A and HNF4 in liver sections from chow diet‐ or HFD‐fed mice (K) and normal individuals or NAFLD patients (L). Nuclei were stained with DAPI. Scale bar, 100 µm. Representative images from four samples per group are shown.

In freshly isolated mouse hepatocytes, the abundance of TMEM16A was comparable with ClC‐2 and 38‐fold approximately higher than CFTR (Figure S1A, Supporting Information), suggesting the importance of TMEM16A Cl^−^ channel in hepatocyte function. A rise in [Ca^2+^]_i_ evoked an outward‐rectifying current (Figure S1B, Supporting Information). The reversal potential (0.7 ± 1.2 mV) was near the equilibrium potential for Cl^−^ (0 mV) under our experimental conditions. This current was significantly decreased by a TMEM16A specific inhibitor or TMEM16A siRNA (**Figure** **1**A,B; and Figure S1C, Supporting Information), indicating that TMEM16A is an essential component of CaCC in hepatocytes. Moreover, HFD and palmitate challenge potentiated this current (Figure 1C,D). To assess whether the increased Ca^2+^‐activated Cl^−^ current (*I*_Cl.Ca_) activity was attributed to TMEM16A upregulation, TMEM16A mRNA and protein levels were examined in liver tissues from mice fed with chow diet or HFD. The expression profile of TMEM16A showed that it was highly distributed in the kidneys and liver (Figure S1D, Supporting Information). A higher expression level of TMEM16A was observed in the livers of mice administered HFD for 32 weeks (Figure 1E,F; and Figure S1E, Supporting Information). The time‐course analysis revealed that TMEM16A expression was gradually increased over the 32 weeks of HFD (Figure 1G). Similarly, TMEM16A expression was significantly upregulated in livers from NAFLD patients compared to those from normal controls (Figure 1H,I; and Figure S1F,G, Supporting Information). Correlation analysis revealed that TMEM16A protein expression was positively correlated with NAFLD score in human subjects (Figure 1J). Interestingly, the abundance of TMEM16A in primary hepatocytes was much higher than that in biliary cells (Figure S1H, Supporting Information), although TMEM16A has also been suggested to be a component of CaCC in biliary epithelial cells.^[^ ^24^ ^]^ Additionally, palmitate treatment markedly increased TMEM16A expression in hepatocytes but not in cholangiocytes (Figure S1I,J, Supporting Information). Immunofluorescence of cross‐sections from mouse and human liver samples further supported the localization of TMEM16A in hepatocytes, which were identified by the hepatocyte marker hepatocyte nuclear factor 4 (HNF4) (Figure 1K,L).

### 2.2 Hepatocyte‐Specific TMEM16A Deletion Ameliorates Obesity and Insulin Resistance

**Figure 2:**
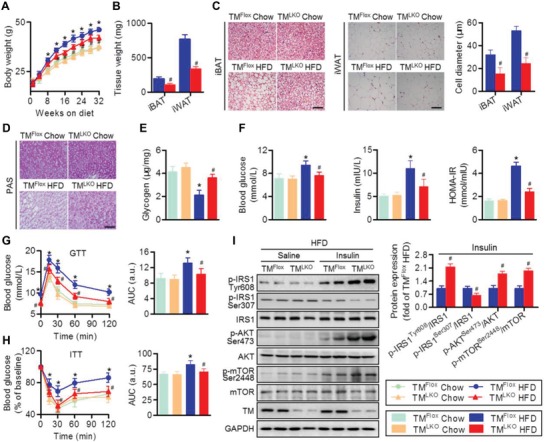
TMEM16A deficiency in hepatocytes inhibits HFD‐induced obesity and insulin resistance. A) Liver‐specific TMEM16A deletion mice (TM^LKO^) and control mice (TM^Flox^) were fed with chow diet or HFD for 32 weeks and then body weight was measured. *P < 0.05 versus TM^Flox^ chow, #*P* < 0.05 versus TM^Flox^ HFD, *n* = 14–18 per group. B,C) Adipose tissue depots iBAT and iWAT were analyzed for weight (B) and adipocyte size (C). Scale bar, 50 µm. #*P* < 0.05 versus TM^Flox^ HFD, *n* = 6 per group. D–F) Hepatic glycogen content (D,E), fasting blood glucose, fasting insulin, and HOMA‐IR index (F) were measured in TM^Flox^ and TM^LKO^ mice on the indicated diet for 32 weeks. Scale bar, 50 µm. *P < 0.05 versus TM^Flox^ chow, #*P* < 0.05 versus TM^Flox^ HFD, *n* = 10 per group. G,H) GTT (G) and ITT (H) were performed on TM^Flox^ mice and TM^LKO^ mice after 32 weeks on chow diet or HFD. *P < 0.05 versus TM^Flox^ chow, #*P* < 0.05 versus TM^Flox^ HFD, *n* = 12 per group. AUC, area under the curve. I) Levels of IRS1 (Tyr608, Ser307), AKT (Ser473), and mTOR (Ser2448) phosphorylation in response to an intraperitoneal injection of insulin (1.0 IU kg^−1^ for 15 min) in the liver tissues of TM^Flox^ and TM^LKO^ mice after HFD treatment. #*P* < 0.05 versus TM^Flox^ insulin, *n* = 6 per group.

The hepatic steatosis‐induced TMEM16A upregulation led us to hypothesize that TMEM16A may alter the development of NAFLD. To this end, we investigated the role of hepatocyte TMEM16A by feeding liver‐specific TMEM16A knockout mice (TM^LKO^) and their control littermates (TM^Flox^) with HFD for 32 weeks (Figure S2A,B, Supporting Information). As expected, hepatic TMEM16A expression and *I*_Cl.Ca_ activity were readily abolished in TM^LKO^ mice but not in TM^Flox^ mice (Figure S2C,D, Supporting Information). TMEM16A ablation in the liver had no effect on the body weight of mice fed a chow diet. However, the HFD‐induced body weight increase was inhibited in TM^LKO^ mice (**Figure** **2**A; and Figure S2E, Supporting Information). Accordingly, the weight and adipocyte diameter of interscapular brown adipose tissue (iBAT) and inguinal white adipose tissue (iWAT) were significantly decreased in HFD‐fed TM^LKO^ mice compared to those in TM^Flox^ mice fed with the same diet, although food intake was similar between mice with different genotypes (Figure 2B,C; and Figure S2F,G, Supporting Information). Periodic acid‐Schiff (PAS) staining and glycogen content measurement revealed that HFD administration induced a marked decrease in glycogen in TM^Flox^ mice, but not in TM^LKO^ mice (Figure 2D,E). Compared to TM^Flox^ mice fed with HFD, TM^LKO^ mice fed with the same diet showed lower fasting blood glucose, insulin, and homeostasis model assessment of insulin resistance (HOMA‐IR) levels (Figure 2F). Moreover, on HFD, TM^LKO^ mice were more glucose tolerant and insulin sensitive compared to TM^Flox^ mice, as determined by intraperitoneal glucose tolerance test (GTT) and insulin tolerance test (ITT) (Figure 2G,H). An acute injection of insulin increased IRS1 (Tyr608), AKT (Ser473), and mTOR (Ser2448) phosphorylation and decreased IRS1 (Ser307) phosphorylation in mouse livers. The activation of the IRS1‐mTOR axis was markedly potentiated in HFD‐fed TM^LKO^ mice (Figure 2I). The ability of insulin to induce this axis activation did not differ between chow diet‐fed TM^LKO^ and TM^Flox^ mice (Figure S2H, Supporting Information).

### 2.3 Hepatocyte‐Specific TMEM16A Overexpression Exacerbates HFD‐Induced Obesity and Insulin Resistance

**Figure 3:**
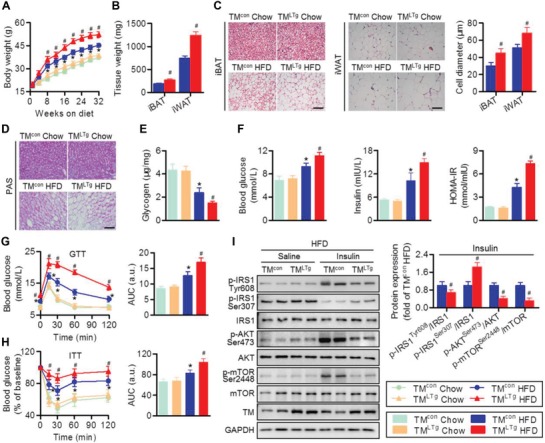
Hepatocyte TMEM16A aggravates HFD‐induced obesity and insulin resistance. A) Body weight of TM^LTg^ and TM^con^ mice on the indicated diets for 32 weeks. *P < 0.05 versus TM^con^ chow, #*P* < 0.05 versus TM^con^ HFD, *n* = 15–20 per group. B) Weight of iBAT and iWAT of TM^con^ and TM^LTg^ mice after HFD treatment. #*P* < 0.05 versus TM^con^ HFD, *n* = 8 per group. C) Representative images of H&E staining of iBAT and iWAT. Adipocyte diameter was calculated. Scale bar, 50 µm. #*P* < 0.05 versus TM^con^ HFD, *n* = 8 per group. D) PAS staining representative images of liver sections of TM^con^ and TM^LTg^ mice on the indicated diets, *n* = 4 per group. Scale bar, 50 µm. E,F) Hepatic glycogen content (E), fasting blood glucose, fasting insulin, and HOMA‐IR index (F) in TM^con^ and TM^LTg^ mice on the indicated diets for 32 weeks. *P < 0.05 versus TM^con^ chow, #*P* < 0.05 versus TM^con^ HFD, *n* = 12 per group. G,H) GTT (G) and ITT (H) were performed on TM^con^ and TM^LTg^ mice after 32 weeks on the indicated diets. *P < 0.05 versus TM^con^ chow, #*P* < 0.05 versus TM^con^ HFD, *n* = 14 per group. I) Phosphorylation of IRS1 (Tyr608, Ser307), AKT (Ser473), and mTOR (Ser2448) in response to an intraperitoneal injection of saline or insulin (1.0 IU kg^−1^ for 15 min) in the liver tissues of TM^con^ and TM^LTg^ mice after being fed with HFD. #*P* < 0.05 versus TM^con^ insulin, *n* = 6 per group.

We subsequently established liver‐specific TMEM16A transgenic mice (TM^LTg^) to further verify the role of TMEM16A in NAFLD (Figure S3A, Supporting Information). Hepatic TMEM16A expression and hepatocyte *I*_Cl.Ca_ activity were greatly increased in TM^LTg^ mice compared to TM^con^ mice (Figure S3B,C, Supporting Information). In contrast to TM^LKO^ mice, TM^LTg^ mice fed with HFD exhibited increased body and visceral fat weight relative to that in HFD‐fed TM^con^ mice (**Figure** **3**A–C; and Figure S3D,E, Supporting Information). Food intake was also similar between TM^con^ mice and TM^LTg^ mice, regardless of HFD challenge (Figure S3F, Supporting Information). The HFD‐induced decrease in glycogen content was more pronounced in TM^LTg^ mice than in TM^con^ mice (Figure 3D,E). Similarly, on HFD, mice overexpressing hepatocyte‐specific TMEM16A displayed higher fasting blood glucose, insulin, and HOMA‐IR levels compared to control littermates (Figure 3F). Moreover, both GTT and ITT results revealed that TM^LTg^ mice were less glucose tolerant and insulin sensitive than TM^con^ mice (Figure 3G,H). Following insulin administration, activation of the IRS1‐mTOR axis in the liver was dramatically inhibited in HFD‐fed TM^LTg^ mice compared to TM^con^ mice fed with the same diet (Figure 3I). The IRS1‐mTOR axis activation did not differ between TM^con^ and TM^LTg^ mice that received a chow diet (Figure S3G, Supporting Information).

### 2.4 TMEM16A Knockdown Inhibits HFD‐Induced Hepatic Steatosis, Lipogenesis, and Inflammation

**Figure 4:**
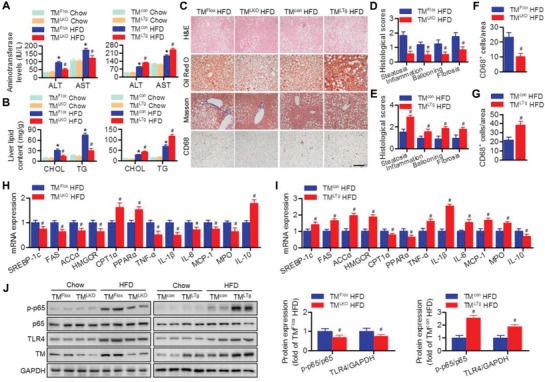
Hepatocyte TMEM16A promotes HFD‐induced hepatic steatosis and inflammation. A,B) Plasma ALT and AST (A), and hepatic cholesterol (CHOL) and triglyceride (TG) (B) levels in TM^LKO^ mice, TM^LTg^ mice, and their control littermates on the indicated diets for 32 weeks. *P < 0.05 versus TM^Flox^ chow or TM^con^ chow, #*P* < 0.05 versus TM^Flox^ HFD or TM^con^ HFD, *n* = 8–12 per group. C–G) Representative H&E, Oil Red O, Masson's trichrome, and CD68 staining of liver sections of TM^LKO^ mice, TM^LTg^ mice, and their control littermates after 32 weeks of HFD (C). Quantification of histological scores (D,E) and CD68‐positive macrophages (F,G). Scale bar, 100 µm. #*P* < 0.05 versus TM^Flox^ HFD or TM^con^ HFD, *n* = 6–8 per group. H–J) mRNA levels of genes associated with cholesterol synthesis, fatty acid synthesis, fatty acid oxidation, and inflammatory response (H,I) as well as p65 phosphorylation and TLR4 protein expression (J) levels in the livers of TM^LKO^ mice, TM^LTg^ mice, and their control littermates fed with the indicated diets for 32 weeks. #*P* < 0.05 versus TM^Flox^ HFD or TM^con^ HFD, *n* = 6 per group.

Insulin resistance can lead to excessive accumulation of lipids and inflammation in the liver, which are key contributors to NAFLD.^[^ ^1^, ^3^, ^4^ ^]^ After the HFD feeding period, the increased liver size and weight, the ratio of liver weight to body weight, and levels of plasma alanine aminotransferase (ALT), aspartate aminotransferase (AST), hepatic cholesterol, and triglyceride were attenuated in TM^LKO^ mice. In contrast, TM^LTg^ mice showed the opposite effects without alteration of the liver weight to body weight ratio (**Figure** **4**A,B; and Figure S4A–D, Supporting Information). Histological analysis of liver sections showed more severe steatosis, lobular inflammation, hepatocyte injury (ballooning), lipid accumulation, hepatic fibrosis, and macrophage infiltration in HFD‐fed TM^LTg^ mice than in TM^con^ mice fed with the same diet. Moreover, HFD‐induced steatohepatitis was markedly ameliorated in TM^LKO^ mice compared to TM^Flox^ mice (Figure 4C–G). Similarly, the hepatic mRNA levels of genes associated with cholesterol and fatty acid synthesis as well as proinflammatory factors were lower, whereas mRNA levels of genes associated with fatty acid oxidation, such as carnitine palmitoyltransferase 1α (CPT1α) and peroxisome proliferator‐activated receptor α (PPARα), and anti‐inflammatory cytokines, such as interleukin (IL)‐10, were higher in HFD‐fed TM^LKO^ mice than in TM^Flox^ mice fed with the same diet (Figure 4H). Hepatocyte‐specific TMEM16A overexpression further increased gene expression associated with cholesterol and fatty acid synthesis and the inflammatory response, while limiting gene expression associated with fatty acid oxidation (Figure 4I). Moreover, HFD‐induced p65 phosphorylation and expression of toll‐like receptor 4 (TLR4) were inhibited in mice with hepatocyte‐specific TMEM16A knockout but enhanced in mice with hepatocyte‐specific TMEM16A overexpression (Figure 4J). Taken together, these results suggest that hepatocyte TMEM16A promotes the development of steatohepatitis.

To determine if our in vivo findings were also reproducible in vitro, we next examined the direct role of TMEM16A in regulating hepatocyte responses. In primary hepatocytes, palmitate markedly increased lipid accumulation, cholesterol content, and triglyceride levels. The increased intracellular lipid deposition was counteracted by TMEM16A deficiency but further enhanced by TMEM16A overexpression (Figure S5A,B, Supporting Information). In bovine serum albumin (BSA)‐treated hepatocytes, neither TMEM16A knockout nor overexpression had an effect on insulin‐induced IRS1‐mTOR axis activation (Figure S5C, Supporting Information). However, following palmitate treatment, TMEM16A knockout potentiated, while TMEM16A overexpression inhibited, IRS1‐mTOR axis activation (Figure S5D, Supporting Information). Palmitate treatment significantly increased p65 phosphorylation and TLR4 expression in hepatocytes; these effects were attenuated by TMEM16A deficiency but further enhanced by TMEM16A overexpression (Figure S5E,F, Supporting Information).

### 2.5 TMEM16A Impairs Glucose Uptake and GLUT2 Translocation

**Figure 5:**
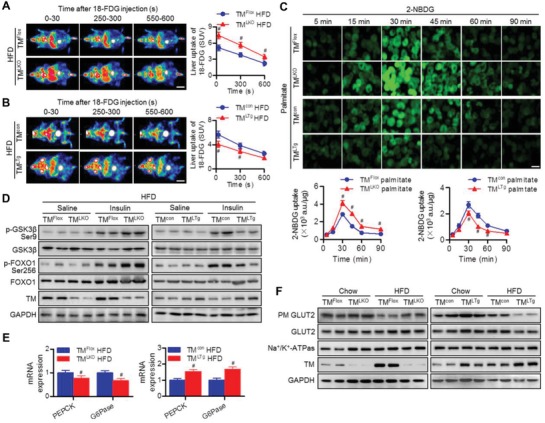
TMEM16A exacerbates HFD‐induced inhibition of hepatic glucose uptake and GLUT2 translocation. A,B) Representative PET images (left panel) and quantification (right panel) of hepatic 18‐FDG uptake in TM^LKO^ mice (A), TM^LTg^ mice (B), and their control littermates after HFD for 32 weeks. SUV, standardized uptake value. Scale bar, 20 mm. #*P* < 0.05 versus TM^Flox^ HFD or TM^con^ HFD, *n* = 5 per group. C) Hepatocytes isolated from TM^LKO^ mice, TM^LTg^ mice, and their control littermates were treated with palmitate for 24 h followed by incubation in culture medium without glucose for 6 h. Afterward, cells were treated with 2‐NBDG (50 µmol L^−1^) for the indicated duration. Representative images of 2‐NBDG fluorescence from four independent experiments. Scale bar, 20 µm. Quantitation of 2‐NBDG fluorescence intensity using a fluorescence microplate reader. #*P* < 0.05 versus TM^Flox^ palmitate or TM^con^ palmitate, *n* = 6. D) Phosphorylation of GSK3β (Ser9) and FOXO1 (Ser256) in response to an intraperitoneal injection of saline or insulin (1.0 IU kg^−1^ for 15 min) in the liver tissues of TM^LKO^ mice, TM^LTg^ mice, and their control littermates after being fed with HFD, *n* = 6 per group. E) mRNA levels of PEPCK and G6Pase in the liver tissues of TM^LKO^ mice, TM^LTg^ mice, and their control littermates fed with HFD for 32 weeks. #*P* < 0.05 versus TM^Flox^ HFD or TM^con^ HFD, *n* = 5. F) TM^LKO^ mice, TM^LTg^ mice, and their control littermates were fed with the indicated diets for 32 weeks. Thereafter, mice were fasted for 12 h and then refed for 4 h. Plasma membrane (PM) and total GLUT2 levels in liver tissues, *n* = 6 per group.

Disturbance of liver glucose uptake manifests as both glucose intolerance and insulin resistance.^[^ ^7^ ^]^ As TM^LKO^ mice displayed decreased hyperglycemia and glucose intolerance, we examined the liver glucose uptake and utilization in TM^LKO^ mice and TM^LTg^ mice. Positron emission tomography/computed tomography (PET/CT) analysis showed that 18‐FDG uptake was increased in the livers of HFD‐fed TM^LKO^ mice compared to those of HFD‐fed TM^Flox^ mice, whereas TM^LTg^ mice were associated with decreased 18‐FDG uptake and metabolic activity (**Figure** **5**A,B). By employing 2‐NBDG, we observed that maximum glucose uptake in hepatocytes was attained after 30 min, followed by a gradual decline over the observed time points. Similarly, TMEM16A overexpression inhibited 2‐NBDG uptake at the indicated time points, while TMEM16A knockdown promoted a significant increase in glucose uptake (Figure 5C). Consistent with the increased glycogen content observed in histological analysis, the level of insulin‐induced phosphorylated glycogen synthase kinase 3 beta (GSK3β) in the liver was higher in TM^LKO^ mice than in TM^Flox^ mice after HFD treatment (Figure 5D). Additionally, TM^LKO^ mice fed with HFD showed reduced mRNA expression of gluconeogenesis enzymes (phosphoenolpyruvate carboxykinase [PEPCK] and glucose‐6‐phosphatase [G6Pase]) and increased phosphorylation of the upstream forkhead box protein O1 (FOXO1) compared to TM^Flox^ mice fed with the same diet. Conversely, hepatocyte‐specific TMEM16A overexpression inhibited GSK3β and FOXO1 phosphorylation but potentiated gluconeogenesis‐related mRNA expression (Figure 5D,E). To unveil the mechanism behind TMEM16A‐potentiated impairment in liver glucose uptake, we determined the hepatic expression of GLUTs. In line with previous studies,^[^ ^7^, ^9^ ^]^ GLUT2 was the major GLUT isoform found in hepatocytes (Figure S6A, Supporting Information). Total GLUT2 levels were similar across all groups of mice; however, HFD induced a marked decrease in plasma membrane GLUT2 levels in the liver, which was attenuated in TM^LKO^ mice but further potentiated in TM^LTg^ mice (Figure 5F). Similarly, TMEM16A knockout inhibited, whereas TMEM16A upregulation enhanced, the palmitate‐induced decrease in plasma membrane GLUT2 levels in isolated hepatocytes (Figure S6B, Supporting Information). These results suggest that TMEM16A is crucial for GLUT2 trafficking and glucose uptake in hepatocytes.

### 2.6 VAMP3 is Involved in TMEM16A‐Mediated GLUT2 Translocation

**Figure 6:**
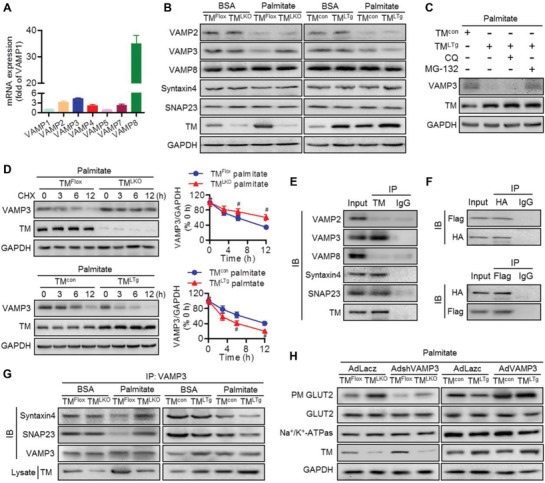
VAMP3 is required for TMEM16A‐mediated GLUT2 translocation. A) Abundance of VAMPs (VAMP1, VAMP2, VAMP3, VAMP4, VAMP5, VAMP7, and VAMP8) in hepatocytes, *n* = 4. B) Protein expression of VAMP2, VAMP3, VAMP8, syntaxin 4, and SNAP23 in hepatocytes from TM^LKO^ mice, TM^LTg^ mice, and their control littermates after BSA or palmitate treatment for 24 h, *n* = 6. C) VAMP3 expression in hepatocytes from TM^con^ mice pretreated with MG‐132 (10 µmol L^−1^) or chloroquine (CQ, 1 µmol L^−1^) for 30 min followed by palmitate treatment for 24 h, *n* = 7. D) Following palmitate treatment, VAMP3 expression was determined in the four groups of hepatocytes pretreated with cycloheximide (CHX, 100 µg mL^−1^) for the indicated durations. #*P* < 0.05 versus TM^Flox^ palmitate or TM^con^ palmitate, *n* = 4. E) Immunoprecipitation (IP) followed by immunoblotting (IB) of hepatocyte lysates showing the presence of VAMP3, syntaxin 4, and SNAP23 in TMEM16A immunoprecipitates, *n* = 4. F) Hepatocytes were cotransfected with VAMP3‐Flag and TMEM16A‐RFP‐HA. Immunoblotting for Flag and HA after immunoprecipitation with HA (upper panel) or Flag (lower panel) antibodies, *n* = 4. G) Immunoblotting analysis of syntaxin 4 and SNAP23 in VAMP3 immunoprecipitates from hepatocytes treated as described, *n* = 6. H) Hepatocytes from TM^LKO^ mice, TM^LTg^ mice, and their control littermates were infected with AdVAMP3, AdshVAMP3, or control adenovirus (AdLacz) for 24 h followed by palmitate treatment. Thereafter, cells were incubated in glucose‐free medium for 6 h and then transferred to high glucose DMEM for 2 h. Distribution of GLUT2 in hepatocytes was analyzed by western blotting, *n* = 6.

The membrane fusion machinery requires the assembly of soluble N‐ethylmaleimide‐sensitive factor attachment protein receptors (SNAREs) comprising vesicle (v)‐SNAREs (vesicle‐associated membrane proteins [VAMPs]) and target membrane (t)‐SNAREs (syntaxin 4 and synaptosome‐associated protein of 23 kDa [SNAP23]).^[^ ^25^, ^26^ ^]^ Although the similarities in the molecular mechanisms underlying the translocation of GLUT2 and GLUT4 to the plasma membrane are unclear, the essential role of SNAREs in regulating GLUT4 translocation^[^ ^8^, ^25^ ^]^ and the location of TMEM16A and GLUT2 in vesicle^[^ ^9^, ^27^ ^]^ led us to ask whether SNAREs are involved in TMEM16A‐mediated GLUT2 translocation. VAMP2, 3, and 8 mRNA levels were highly expressed in hepatocytes in comparison to VAMP1 mRNA, and VAMP8 levels were 10.9‐ and 8.2‐fold higher than those of VAMP2 and VAMP3, respectively (**Figure** **6**A). Palmitate markedly decreased the expression of VAMP2 and VAMP3 in hepatocytes. Only the decrease in VAMP3 expression was reversed by TMEM16A deficiency and potentiated by TMEM16A upregulation. The reduced VAMP2 expression remained unchanged. Additionally, the expression of VAMP8, syntaxin 4, and SNAP23 was comparable among the different groups (Figure 6B). TMEM16A knockout or overexpression did not alter the palmitate‐induced decrease in VAMP3 mRNA levels (Figure S7A,B, Supporting Information). Meanwhile, the effect of TMEM16A overexpression on VAMP3 protein expression was restored by proteasome inhibitor MG132, but not lysosome blocker chloroquine (Figure 6C). By using a protein synthesis inhibitor cycloheximide, the degradation of VAMP3 was markedly inhibited by TMEM16A knockout but enhanced by TMEM16A overexpression (Figure 6D), suggesting that TMEM16A inhibits VAMP3 expression by enhancing VAMP3 degradation through the proteasome‐dependent pathway. Immunoprecipitation assays showed that TMEM16A coprecipitated with VAMP3, syntaxin 4, and SNAP23 but not with VAMP2 or VAMP8 (Figure 6E). Consistent with this, TMEM16A was also found to exogenously interact with VAMP3 in hepatocytes transfected with HA‐RFP‐TMEM16A and Flag‐VAMP3 (Figure 6F).

Taking into consideration the association between TMEM16A and VAMP3, syntaxin 4, and SNAP23, we examined whether TMEM16A affects SNAREs assembly. TMEM16A overexpression significantly limited the formation of VAMP3/syntaxin 4 and VAMP3/SNAP23 complexes, whereas TMEM16A deficiency led to the opposite effect (Figure 6G), suggesting that degradation of VAMP3 by TMEM16A contributes to the impairment of SNAREs formation. To clarify the pathophysiological relevance of VAMP3 in TMEM16A‐mediated GLUT2 translocation, hepatocytes from TM^LKO^ or TM^LTg^ mice were infected with VAMP3 shRNA adenovirus (AdshVAMP3) or VAMP3 adenovirus (AdVAMP3) and their control adenovirus, respectively (Figure S7C,D, Supporting Information). VAMP3 knockdown markedly decreased the plasma membrane GLUT2 level in hepatocytes from both TM^Flox^ mice and TM^LKO^ mice, whereas overexpression of VAMP3 abrogated the inhibitory effect of TMEM16A upregulation on the plasma membrane GLUT2 level (Figure 6H). Collectively, these findings indicate that VAMP3 degradation and the subsequent impairment of SNAREs formation underlie, at least in part, the deleterious effects of TMEM16A on GLUT2 translocation and glucose uptake.

### 2.7 VAMP3 is Indispensable for TMEM16A Function in Hepatocytes

**Figure 7:**
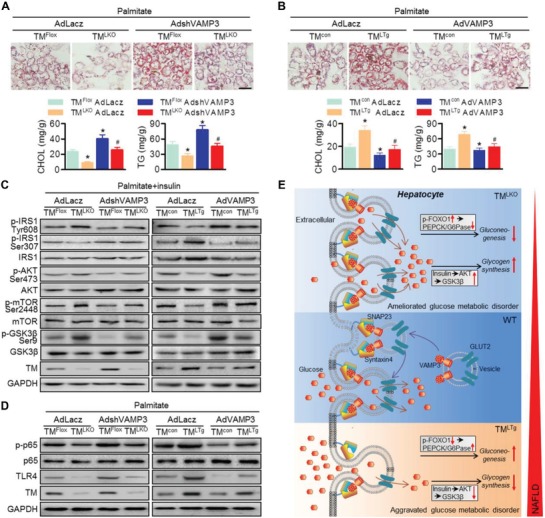
Restoration of VAMP3 abrogates the deleterious effects of TMEM16A on hepatocyte function. A,B) Hepatocytes from TM^LKO^ mice, TM^LTg^ mice, and their control littermates were infected with AdVAMP3 (A), AdshVAMP3 (B), or AdLacz for 24 h prior to palmitate stimulation. Representative images of Oil Red O staining. Cholesterol content and triglyceride levels were measured in hepatocytes. Scale bar, 20 µm. *P < 0.05 versus TM^Flox^ AdLacz or TM^con^ AdLacz, #*P* < 0.05 versus TM^LKO^ AdLacz or TM^LTg^ AdLacz, *n* = 5. C) Phosphorylation of IRS1 (Tyr608, Ser307), AKT (Ser473), and mTOR (Ser2448) in response to insulin (100 nmol L^−1^) stimulation for 30 min, *n* = 6. D) p65 phosphorylation and TLR4 protein expression, *n* = 6. E) Schematic representation of the study findings. TMEM16A binds to VAMP3 and induces VAMP3 degradation, inhibiting VAMP3/syntaxin 4 and VAMP3/SNAP23 complex formation in hepatocytes. As such, TMEM16A insufficiency promotes formation of these complexes and, consequently, GLUT2 translocation, leading to enhanced glucose uptake and glycogen synthesis as well as decreased insulin resistance and gluconeogenesis. This coordinately ameliorates glucose metabolic disorder and other NAFLD‐related events.

Accordingly, to investigate whether VAMP3 is required for the regulatory function of hepatocyte TMEM16A in lipid accumulation, insulin signaling, and inflammatory response, we compared hepatocytes from TM^LKO^ and TM^LTg^ mice treated with AdshVAMP3 or AdVAMP3 in the presence of palmitate. VAMP3 knockdown markedly potentiated the increase of lipid accumulation, cholesterol content, and triglyceride levels in hepatocytes isolated from TM^Flox^ and TM^LKO^ mice, while the enhanced effect of TMEM16A overexpression on lipid deposition was abrogated by AdVAMP3 (**Figure** **7**A,B). Compared to TMEM16A‐deficient hepatocytes treated with Ad‐Lacz, treatment with AdshVAMP3 attenuated IRS1‐GSK3β axis activation, which was potentiated by TMEM16A ablation. However, VAMP3 upregulation offset the inhibitory effects of TMEM16A overexpression on the IRS1‐GSK3β axis (Figure 7C). Additionally, knockdown of VAMP3 in TMEM16A‐deficient hepatocytes recovered p65 phosphorylation and TLR4 expression that were inhibited by TMEM16A deficiency. However, the more pronounced p65 phosphorylation and TLR4 expression in TM^LTg^ hepatocytes were suppressed by AdVAMP3 (Figure 7D).

## 3 Discussion

In this study, we provide evidence that TMEM16A plays a critical role in NAFLD by employing liver‐specific TMEM16A transgenic or deletion mice. In response to a prosteatotic stimulus, such as HFD, TMEM16A expression is increased and it binds to VAMP3, resulting in degradation of the latter and disruption of SNAREs formation. The impairment of this complex formation limits GLUT2 translocation to the plasma membrane and glucose uptake in hepatocytes. Suppression of glucose uptake by TMEM16A also generates secondary effects by enhancing gluconeogenesis and glucose metabolic disorder, thereby exacerbating insulin resistance, lipogenic events, inflammatory responses, and other NAFLD‐related events (Figure 7E).

In line with a previous study in guinea‐pig hepatocytes,^[^ ^23^ ^]^ our results also support the existence of CaCCs in mouse hepatocytes. Furthermore, our results demonstrate that TMEM16A is responsible for CaCC activation in hepatocytes, since the *I*_Cl.Ca_ can be abrogated by TMEM16A inhibition. Moreover, TMEM16A expression was increased in liver tissues from patients and mice with NAFLD. The increased abundance of TMEM16A in the liver was accompanied by an increase in NAFLD score, suggesting a key role of TMEM16A in hepatic steatosis. We further demonstrated that hepatocyte TMEM16A aggravated hepatic glucose metabolic disorder, steatosis, and inflammation. In addition to the deleterious effects on hepatic physiological processes, TMEM16A also exerted systemic effects including obesity, adipocyte hypertrophy, visceral fat weight gain, glucose intolerance, and insulin hyposensitivity, which may be attributed primarily to liver dysfunction.

The increased glucose tolerance in TM^LKO^ mice seems to contrast with the observations in TMEM16A global knockdown mice, in which a more severe glucose intolerance, due to deficiency of insulin secretion at the early stage of HFD (5 weeks) was reported.^[^ ^22^ ^]^ An in vitro study further suggested that TMEM16A is required for glucose‐induced insulin secretion in β cells.^[^ ^21^ ^]^ Two factors may explain this discrepancy. First, in a study by Xu and co‐workers,^[^ ^22^ ^]^ differences in blood glucose between wild‐type and heterozygous mice were diminished after 13 weeks of HFD administration, while the inhibitory effect of TMEM16A knockdown on insulin secretion was long‐lasting. This suggests that ectopic overexpression of TMEM16A may cause hyperinsulinemia, accompanied by a transition from simple hyperlipidemia to insulin resistance and, subsequently, NAFLD. In fact, our results were consistent with the latter possibility, as we found higher insulin levels and hyposensitivity to insulin in TM^LTg^ mice fed with HFD for 32 weeks. Furthermore, the tissue distribution of TMEM16A is heterogeneous; this may be directly associated with the effects of TMEM16A global knockdown on insulin secretion regulation in β cells, as this is unlikely to happen when TMEM16A is specifically knocked out in the liver.

The involvement of TLR4 in palmitate‐induced inflammatory responses has been reported in macrophages and adipocytes.^[^ ^1^, ^5^ ^]^ Although the majority of the studies that have linked TLR4 activation to NAFLD have focused on macrophages, emerging evidence suggests that hepatocyte TLR4 is an attractive target for NAFLD therapy.^[^ ^1^, ^2^, ^28^, ^29^, ^30^ ^]^ Upregulation of TLR4 was observed in NASH patients and murine dietary models.^[^ ^2^, ^29^ ^]^ Hepatocyte‐specific knockout of TLR4 showed ameliorated glucose and insulin intolerance as well as steatohepatitis in HFD‐fed mice.^[^ ^28^ ^]^ The activation of TLR4 in NASH pathologies is primarily mediated by the nuclear factor κ‐light‐chain‐enhancer of activated B cells (NF‐κB)/p65 cascade, which plays a critical role in regulating hepatic inflammation and insulin resistance.^[^ ^1^, ^2^, ^29^ ^]^ Interestingly, our previous work revealed that NF‐κB signaling can be mediated by Cl^−^ channels.^[^ ^31^ ^]^ Consistent with this, hepatocyte TMEM16A overexpression potentiated the activation of the NF‐κB‐TLR4 axis, whereas TMEM16A knockout exerted the opposite effect.

Hepatic glucose uptake and utilization are indispensable for maintaining systemic glucose homeostasis.^[^ ^6^, ^30^ ^]^ In this study, TMEM16A deletion increased hepatic glucose uptake. Additionally, the increased glucose uptake in HFD‐fed TM^LKO^ mice was followed by an increase in hepatic glycogen synthesis via GSK3β phosphorylation and a decrease in gluconeogenesis via inhibition of FOXO1/PEPCK/G6Pase axis activation. This suggests that deficiency of TMEM16A facilitates glucose uptake and improves hyperglycemia, one of the main features of insulin‐resistant conditions. Compromised glucose uptake and conversion to glycogen may disturb hepatic glucose sensing and contribute to hepatic steatosis via the DNL pathway.^[^ ^6^, ^7^ ^]^ Supporting this notion, HFD‐fed TM^LTg^ mice with a more severe glucose metabolic disorder presented with significantly exacerbated steatohepatitis. Moreover, in contrast to adipocytes and smooth muscle cells, hepatic glucose uptake is primarily mediated by GLUT2, not GLUT4, in hepatocytes.^[^ ^9^ ^]^ Here, TMEM16A overexpression further potentiated the inhibition of GLUT2 translocation to the plasma membrane in response to HFD or palmitate challenge, which was consistent with the decrease in glucose uptake. These findings suggest that the defective translocation of GLUT2 may account for the impaired glucose uptake and consequent glucose metabolic disorder in HFD‐fed TM^LTg^ mice. The inhibition of GLUT2‐mediated glucose uptake in TM^LTg^ mice was analogous to that in liver‐specific GLUT2 knockout mice, in which animals showed suppressed hepatic glucose uptake but not glucose secretion, resulting in glucose intolerance and insulin insensitivity.^[^ ^7^ ^]^

Notably, TMEM16A has been shown to physically interact with SNARE proteins, such as VAMP3 and syntaxin 4, which are integral to translocation of vesicles to the plasma membrane, in particular for GLUT4 membrane docking and fusion.^[^ ^8^, ^16^, ^25^, ^32^ ^]^ The present study reveals that SNARE proteins are also involved in GLUT2 translocation to the plasma membrane. VAMP2, VAMP3, and VAMP8 have been found to regulate exocytosis in different cell types via interaction with t‐SNARE proteins, such as syntaxin 4 and SNAP23.^[^ ^25^, ^32^, ^33^ ^]^ In hepatocytes, the level of VAMP8 was strikingly higher than that of VAMP2 and VAMP3. Nonetheless, only VAMP3 expression was affected by TMEM16A under palmitate treatment conditions. Surprisingly, TMEM16A had no effect on the mRNA level of VAMP3 but promoted its protein degradation. Similar to a previous study,^[^ ^16^ ^]^ immunoprecipitation of TMEM16A coprecipitated VAMP3 and syntaxin 4 but not VAMP2 and VAMP8 in hepatocytes. Additionally, we further identified the interaction between TMEM16A and SNAP23 and found that TMEM16A indeed influences the assembly of the SNARE complex, including VAMP3, syntaxin 4, and SNAP23. Thus, the ability of TMEM16A to attenuate SNARE complex formation is closely associated with the degradation of VAMP3.

Importantly, restoration of VAMP3 offset the inhibitory effect of TMEM16A on GLUT2 translocation, suggesting that VAMP3 is a major v‐SNARE involved in GLUT2 trafficking in hepatocytes. However, how is this observation related to the aggravated steatohepatitis in TM^LTg^ mice? Our results showed that VAMP3 overexpression remarkably reversed the effect of TMEM16A in promoting hepatocyte lipid accumulation, insulin resistance, and inflammation. These data are consistent with those from a previous work that suggests a role for VAMP3 in the amelioration of cardiac insulin resistance,^[^ ^8^ ^]^ indicating that VAMP3 is important for the regulation of glucose and insulin tolerance. Intriguingly, according to a report by Yang et al.,^[^ ^26^ ^]^ global knockout of VAMP3 results in normal insulin levels and glucose tolerance. There is no clear explanation for the different outcomes of VAMP3 knockdown and ablation. Since the function of VAMP2, VAMP3, or VAMP8 alone was sufficient to maintain insulin and glucose homeostasis,^[^ ^25^, ^32^ ^]^ the compensatory effects of alternative v‐SNARE proteins are unknown under complete ablation of VAMP3 during muscle and adipose tissue development. Moreover, despite the fact that VAMP3 absence does not affect glucose uptake in adipocytes and skeletal muscle cells,^[^ ^6^ ^]^ similar data on the liver, where it is essential to maintain glucose homeostasis, do not yet exist. Thus, specific modulation of VAMP3 levels may be more relevant for steatohepatitis treatment.

## 4 Conclusion

The present study demonstrated that hepatocyte TMEM16A aggravates hepatic glucose metabolic disorder, steatosis, insulin resistance, and inflammation induced by HFD, thus promoting the development of NAFLD. Mechanistically, the prosteatotic role of TMEM16A is mediated by VAMP3. These findings suggest that development of stable small molecules that targets hepatocyte TMEM16A or screening an inhibitor that blocks TMEM16A/VAMP3 interaction may be a viable therapeutic strategy for NAFLD treatment.

## 5 Experimental Section

##### Antibodies and Reagents

The following antibodies were purchased from Cell Signaling Technology (Danvers, MA), p‐IRS1 (Ser307; #2381; 1:1000), IRS1 (#2382; 1:1000), p‐AKT (Ser473; #4060; 1:1000), AKT (#4691; 1:1000), p‐mTOR (Ser2448; #5536; 1:1000), mTOR (#2983; 1:1000), p‐p65 (#3033; 1:500), p65 (#8242; 1:500), p‐GSK3β (Ser9; #5558; 1:1000), GSK3β (#12 456; 1:1000), FOXO1 (#2880; 1:1000), VAMP2 (#13 508; 1:250), VAMP3 (#13 640; 1:1500), VAMP8 (#13 060; 1:500), GADPH (#97 166; 1:4000), HA‐Tag (#3724; 1:1000), Flag‐Tag (#14 793; 1:1000), HRP‐linked anti‐mouse IgG (#7076; 1:1000), and HRP‐linked antirabbit IgG (#7074; 1:1000). Antibodies against TLR4 (sc‐293072; 1:1000), SNAP23 (sc‐166244; 1:1000), syntaxin 4 (sc‐101301; 1:500), and Na^+^/K^+^‐ATPase (sc‐58629; 1:1000) were obtained from Santa Cruz Biotechnology (Dallas, TX). p‐IRS1 antibody (Tyr608; 09–432; 1:1000) was purchased from Millipore (Burlington, MA). Antibodies against TMEM16A (ab53212; 1:2000 for western blotting, 1:100 for immunohistochemistry and immunofluorescence), HNF4 (ab41898; 1:100), CD68 (ab125212; 1:50), p‐FOXO1 (Ser256; ab131339; 1:1000), and GLUT2 (ab54460; 1:500) were purchased from Abcam (Cambridge, UK). Unless otherwise indicated, all chemicals were purchased form Sigma‐Aldrich (St. Louis, MO).

##### Animal Model

Conditional deletion of TMEM16A was established using the Cre/*lox*P recombination by Cyagen (Suzhou, China). The genotyping strategy is illustrated in Figure S2A (Supporting Information). Mouse genomic fragments containing homology arms and a conditional knockout (CKO) region were generated by polymerase chain reaction (PCR) using a bacterial artificial chromosome (BAC) clone from the C57BL/6J library as a template. The targeting vector was designed for CKO by flanking TMEM16A exon 12 with two *lox*P sites and introducing a neo selection cassette flanked by Frt sites that can remove 53 amino acids of the second transmembrane domain as well as an extracellular loop between the first and the second transmembrane domains. The construct was linearized and introduced into 129 Sv/J embryonic stem cells (ESCs), which were identified by neomycin selection. ESC clones were confirmed by Southern blotting and then microinjected into blastocysts derived from C57BL/6 J mice. The injected blastocysts were then transferred into pseudopregnant female mice and the chimeric males were identified by the presence of agouti hair. After removal of the resistance cassette, the offspring were successfully backcrossed to TMEM16A^Flox^ (TM^Flox^) homozygosity. Albumin‐Cre transgenic mice were purchased from the Jackson Laboratory (Bar Harbor, ME) and then backcrossed to a C57BL/6 genetic background for at least nine generations. Liver‐specific TMEM16A knockout mice (TM^LKO^) were generated by crossing TM^Flox^ with Albumin‐Cre mice. Mice were genotyped by PCR using two pairs of flox primers and one pair of Cre primer, floxed region 1 forward, 5′‐GGTATCACCCAAGGTAACCATCCA‐3′ and reverse, 5′‐CAACCCTCTCTATCCCTGTCACATG‐3′; floxed region 2 forward, 5′‐TGATTCTGATAGCAAATGAGGCAGAT‐3′ and reverse, 5′‐AGGTTATCATAGCTCAGTTCACAAGCTTT‐3′; Albumin‐Cre forward, 5′‐GAAGCAGAAGCTTAGGAAGATGG‐3′ and reverse, 5′‐TTGGCCCCTTACCATAACTG‐3′.

TMEM16A transgenic mice were generated by Cyagen as previously described.^[^ ^19^ ^]^ Briefly, a transgene construct containing TMEM16A cDNA was inserted into the pRP.ExBi‐CMV‐*Lox*P‐Stop‐*Lox*P cassette and microinjected into fertilized mouse embryos. In this strain (referred to as TM^con^), TMEM16A was not overexpressed because of the “stop codon” system. Liver‐specific TMEM16A transgenic mice (TM^LTg^) were created by crossing the Albumin‐Cre mice. Mice were genotyped by PCR using the following primers, *lox*P site forward, 5′‐TCATGTCTGGATCCCCATCAAGC‐3′ and reverse, 5′‐GAGTACTTCTCGGGGACCCTCA‐3′; and Cre primers (see the above‐mentioned Albumin‐Cre for the primer sequences).

Male mice (8‐week‐old), were fed with chow diet or HFD (60% kcal fat; D12492; Research Diets, Inc., New Brunswick, NJ) for 32 weeks. All animal experiments were performed according to the policies of the Sun Yat‐Sen University Committee for Animal Research and conformed to the Guide for the Care and Use of Laboratory Animals of the National Institute of Health in China.

##### Human Liver Samples

Normal and NAFLD liver tissues were obtained from healthy individuals or patients with NAFLD undergoing biopsy or transplantation in the First Affiliated Hospital of Sun Yat‐Sen University (Guangzhou, China). Patients were recruited for this study after exclusion of significant alcohol consumption, autoimmune liver disease, viral hepatitis, hemochromatosis, and chronic inflammatory bowel diseases. The NAFLD samples were assessed and scored by two experienced histopathologists who were blinded to the clinical data according to the NAFLD scoring system:^[^ ^34^ ^]^ steatosis (0–3), lobular inflammation (0–3), and ballooning (0–2). Clinical and histologic characteristics of these samples are summarized in Table S1 (Supporting Information). All procedures were approved by the Medical Research Ethics Committee of Sun Yat‐Sen University. Written informed consent was obtained from all subjects and the experiments were conducted according to the principles outlined in the Declaration of Helsinki.

##### Isolation of Primary Hepatocytes and Cell Culture

Primary hepatocytes were isolated from mice by liver perfusion. Mice were anesthetized with 2% pentobarbital sodium and the peritoneal cavity was opened. Livers were perfused with Hanks' Balanced Salt Solution (HBSS) containing 5 mmol L^−1^ CaCl_2_, 0.1 mg mL^−1^ DNaseI, and 100 mmol L^−1^ HEPES for 5 min through the portal vein, followed by a second perfusion with collagenase buffer containing 0.5 mg mL^−1^ collagenase, 66.7 mmol L^−1^ NaCl, 6.7 mmol L^−1^ KCl, 50  mmol L^−1^ HEPES, and 4.8  mmol L^−1^ CaCl_2_ for another 5 min at a rate of 5 mL min^−1^. The livers were dissected and gently minced with forceps. The cell suspensions were filtered through a 70 µm nylon cell strainer (BD Falcon, BD Biosciences, San Jose, CA). After centrifugation at 50 × *g* for 2 min, pellets were resuspended in HBSS and centrifuged for 10 min at 200 × *g* using 90% Percoll. The obtained hepatocytes were resuspended in Dulbecco's modified Eagle's medium (DMEM) supplemented with 10% fetal bovine serum, 100 U mL^−1^ penicillin, and 100 U mL^−1^ streptomycin. After determining cell viability via trypan blue exclusion assays, cells were incubated at 37 °C for 24 h prior to all experiments. Nonadherent cells were removed, after which fresh media were added.

##### *I*_Cl.Ca_ Recording

*I*_Cl.Ca_ was recorded using whole‐cell patch with an Axopatch 200B patch‐clamp amplifier (Axon Instruments, Foster City, CA) as described previously.^[^ ^17^, ^19^ ^]^ Patch pipettes were made from borosilicate glass with a Sutter P‐97 horizontal puller (Sutter Instrument Co., Novato, CA). The resistance of the pipettes was 3–6 MΩ after filling with pipette solution. Currents were filtered at 2 kHz and sampled at 5 kHz using pCLAMP8.0 software (Axon Instruments). In experiments, the test potentials were applied from −100 to +100 mV in +20 mV increments for 500 ms with an interval of 5 s. The extracellular solution contained 125 mmol L^−1^ *N*‐*methyl*‐*D*‐glucamine (NMDG)‐Cl, 5 mmol L^−1^ KCl, 1.5 mmol L^−1^ CaCl_2_, 1 mmol L^−1^ MgSO_4_, 10 mmol L^−1^ HEPES, and 10 mmol L^−1^ glucose at pH adjusted to 7.4 with NMDG. The pipette solution contained 130 mmol L^−1^ CsCl, 1 mmol L^−1^ Mg·ATP, 1.2 mmol L^−1^ MgCl_2_, 10 mmol L^−1^ HEPES, 2 mmol L^−1^ EGTA, and 1.639 mmol L^−1^ CaCl_2_ at pH adjusted to 7.4 with CsOH. The [Ca^2+^]_i_ was 500 nmol L^−1^.

##### Molecular Assays

To determine mRNA levels, total RNA was isolated from liver tissues or hepatocytes using TRIzol reagent according to manufacturer's instructions. RNA (2 µg) was reverse transcribed into cDNA using the QuantiTect Reverse Transcription Kit (Qiagen, Hilden, Germany). Real‐time PCR was performed using SYBR Green PCR Master Mix (Invitrogen, Carlsbad, CA) on a MyiQ Single Color Real‐Time PCR Detection System (Bio‐Rad Laboratories, Hercules, CA). The sequence‐specific primers for the genes are listed in Table S2 (Supporting Information) and were synthesized by Invitrogen. The fold change in expression of each gene was calculated using the 2^−ΔΔCT^ method, with glyceraldehyde‐3‐phosphate dehydrogenase as an internal control.

Western blots were performed as described previously.^[^ ^11^, ^12^ ^]^ In brief, protein extracts were prepared from frozen tissues or primary hepatocytes in radioimmunoprecipitation assay lysis buffer supplemented with protease inhibitor cocktail (Merck, Darmstadt, Germany). Cytoplasmic and membrane fractions were extracted using a Qproteome Cell Compartment Kit (Qiagen) according to manufacturer's instructions. Protein content was quantified using the BCA Assay Kit (Thermo Fisher Scientific, Waltham, MA). Protein samples were separated by 6–12% sodium dodecyl sulfate‐polyacrylamide gel electrohoresis and transferred to polyvinylidene fluoride membranes (Millipore). The membranes were blocked in nonfat dry milk for 1 h at room temperature and then incubated with primary antibodies at 4 °C overnight. After incubation with the corresponding secondary HRP‐conjugated antibodies for 1 h at room temperature, HRP was detected with an ECL Kit (Thermo Fisher Scientific) and quantified with ImageJ software (NIH, Bethesda, MD).

##### Histological Analysis

Liver tissues or adipose tissues were fixed in 4% paraformaldehyde, dehydrated, and embedded in Tissue‐Tek OCT compound (Sakura, Tokyo, Japan) or paraffin. Histology was performed on paraffin‐embedded liver tissue sections by staining them with hematoxylin and eosin (H&E), PAS, and Masson's trichrome according to standard procedures, followed by counterstaining with hematoxylin. For immunohistochemical staining of TMEM16A and CD68, liver sections were incubated with primary antibodies against TMEM16A and CD68 overnight at 4 °C and then stained with biotinylated secondary antirabbit antibody at room temperature for 1 h, followed by visualization with 3,3‐diaminobenzidine tetrachloride and counterstaining with hematoxylin. Liver cryosections were stained using Oil Red O to observe lipid accumulation in the liver. Paraffin‐embedded adipose sections were stained with H&E. All images were acquired using a light microscope (IX71; Olympus, Tokyo, Japan). The mean diameter of adipocytes was calculated using ImageJ software.

##### Blood Chemistry

Fasting blood glucose and insulin were measured in 12 h overnight‐fasted mice using a glucometer (OneTouch Ultra Test Strips; Lifescan, Malvern, PA) and an Insulin ELISA Kit (Millipore). The HOMA‐IR index was calculated using the following equation, [fasting blood glucose (mmol L^−1^) × fasting insulin (mIU L^−1^)]/22.5. GTT and ITT were monitored in mice fasted for 12 and 6 h, respectively. For GTT, mice were intraperitoneally injected with 1.5 g kg^−1^ glucose, while 0.5 IU kg^−1^ insulin was intraperitoneally injected into mice for ITT. Blood glucose was tested at 30, 60, 90, and 120 min after injection. Plasma ALT and AST were measured spectrophotometrically by a fully automated clinical chemistry analyzer (BS‐800M; Mindray, Shenzhen, China).

##### Determination of Biochemistry in Liver Tissues

Liver homogenates were prepared in a tenfold volume v/w of isopropyl alcohol and then supernatants were collected after centrifugation at 12 000 × *g* and 4 °C for 15 min. Liver cholesterol content was examined as previously described.^[^ ^12^, ^35^ ^]^ In brief, liver tissues were washed with phosphate‐buffered saline (PBS) and harvested in isopropyl alcohol followed by ultrasonication. Sonicates (0.1 mL) were added to assay solutions (0.9 mL); 0.1 U mL^−1^ cholesterol oxidase, 0.01 U mL^−1^ cholesterol ester hydrolase, 1 U mL^−1^ peroxidase, 0.05% Triton X‐100, 1 mmol L^−1^ sodium cholate, and 0.6 mg mL^−1^ β‐hydroxyphenylacetic acid; pH 7.4) and incubated at 37 °C for 1 h. Fluorescence of the mixture was detected by a fluorospectrometer (RF‐5000; Shimadzu Co., Tokyo, Japan). Triglycerides were determined using the Triglyceride Kit (Wako Diagnostics, Richmond, VA). Total glycogen level was measured with the Glycogen Assay Kit (BioVision, Irvine, CA) according to the manufacturer's instructions. Hepatic cholesterol, triglycerides, and glycogen were normalized to the amount of protein.

##### Measurement of Intracellular Lipid Deposition

Primary hepatocytes treated with siRNA or adenovirus were incubated with BSA or palmitate for 24 h. Cells were then fixed with 4% formaldehyde and stained with Oil Red O to measure intracellular fat content. Cell homogenates were collected for intracellular cholesterol content determination using the same method as described for liver tissues. The fluorescence intensity was normalized to cell protein concentration. Intracellular triglyceride levels were examined using a commercial kit from Cayman (Ann Arbor, MI).

##### siRNA, Plasmid Transfection, and Adenovirus Infection

To knockdown TMEM16A expression, cells were transfected with a siRNA against the mouse TMEM16A gene (5′‐CUGCUCAAGUUUGUGAACUTT‐3′) or negative control siRNA using HiPerfect Transfection Reagent (Qiagen) according to manufacturer's instructions. TMEM16A cDNA (a gift from Dr. L. Y. Jan, University of California, CA) was tagged with RFP‐HA and subcloned into pMSCV using the overlap extension PCR cloning method. The plasmid was then transfected into hepatocytes with Lipofectamine 2000 reagent (Invitrogen) according to manufacturer's instructions. For VAMP3 gain‐ or loss‐of‐function studies, cells were infected with recombinant adenovirus expressing a Flag‐tagged full‐length mouse VAMP3 gene (AdVAMP3) or an shRNA targeting VAMP3 (AdshVAMP3; Cyagen) for 24 h. The sense strand of the shRNA used to knock down VAMP3 gene was 5′‐ATGAAACTGAAGCCCGATATTCTCGAGAATATCGGGCTTCAGTTCAT‐3′. The Lacz adenovirus (AdLacz) was used as negative control.

##### PET/CT Imaging

Before PET/CT imaging, mice were fasted for 12 h and anesthetized with 1.5% isoflurane. Anesthetized mice were injected intravenously with 18‐FDG (150 µCi) via the tail vein. PET acquisitions were performed immediately after 18‐FDG injection. The images were acquired for 10 min using an Inveon multimodality PET/CT system and reconstructed using the 3D maximum expectation maximization method (Inveon Image Research Workplace; Siemens Healthcare, Erlangen, Germany). The standardized uptake value (SUV) for the region of interest was measured by dividing the 18‐FDG activity by the injected dose and animal weight using the Inborn Research Workplace Software provided by the manufacturer.

##### Glucose Uptake Measurement

Glucose uptake of primary hepatocytes was assessed using the fluorescent glucose analog 2‐NBDG (Tocris Bioscience, Bristol, UK). 24 h after palmitate treatment, cells were incubated with glucose‐free DMEM for 6 h, followed by treatment with 2‐NBDG (50 µmol L^−1^) in Krebs‐Ringer‐Bicarbonate buffer (129 mmol L^−1^ NaCl, 4.7 mmol L^−1^ KCl, 1.2 mmol L^−1^ KH_2_PO_4_, 1.0 mmol L^−1^ CaCl_2_, 1.2 mmol L^−1^ MgSO_4_, 5.0 mmol L^−1^ NaHCO_3_, and 10 mmol L^−1^ HEPES; pH 7.4) at increasing durations ranging from 5 to 90 min. For each incubation period, cells were washed twice with PBS to stop the uptake reaction and imaged immediately using a fluorescent microscope (IX71). To quantify the fluorescence intensity of 2‐NBDG, hepatocytes were seeded in black 96‐well plates and subjected to the aforementioned treatment. Fluorescence was measured using a fluorescence microplate reader (Infinite F500; Mannedorf, Switzerland) and normalized to protein concentration in each well.

##### Immunoprecipitation Assay

Immunoprecipitation was performed as previously described.^[^ ^11^ ^]^ Cell lysates were adjusted to equal amounts of protein (500 µg) and immunoprecipitated with TMEM16A, HA, Flag, or VAMP3 antibodies at 4 °C overnight. Rabbit IgG control antibody (#3423; 1:50; Cell Signaling Technology) was used as a negative control. Immunoprecipitates were pulled down with Protein PLUS A/G Agarose (sc‐2003; Santa Cruz Biotechnology) followed by western blot analysis.

##### Confocal Immunofluorescence Microscopy

For immunofluorescence staining of TMEM16A and HNF4 in liver sections, the sections were blocked with 5% BSA, followed by incubation with primary antibodies at 4 °C overnight. After three washes with PBS, sections were incubated with Cy3‐labeled antirabbit IgG (#A10520; 1:100; Invitrogen) and FITC‐labeled antimouse IgG (#62‐6511; 1:100; Invitrogen) for 1 h. Images were captured using a confocal microscope (Zeiss LSM800; Zeiss, Oberkochen, Germany).

##### Statistical Analysis

All data are expressed as the mean ± standard error of the mean (SEM). For patch‐clamp studies, *n* represents the number of recorded cells from different independent batches of cells. For other studies, *n* represents the number of independent experiments performed on different mice or different batches of cells. Statistical analysis was performed using SPSS 16.0 software (SPSS Inc., Chicago, IL) and unpaired two‐tailed Student's *t*‐test or one‐way analysis of variance (ANOVA) followed by Bonferroni's multiple comparisons post hoc test with a 95% confidence interval. Correlation analyses were performed using the Pearson correlation test. *P* < 0.05 was considered statistically significant.

## Conflict of Interest

The authors declare no conflict of interest.

## Supporting information

Supporting Information
