# Supplementary material for: Hepatocyte TMEM16A Deletion Retards NAFLD Progression by Ameliorating Hepatic Glucose Metabolic Disorder
Source: Adv Sci (Weinh). 2020 Mar 20;7(10):1903657. doi: 10.1002/advs.201903657 (PMC7237841; doi:10.1002/advs.201903657)
Supplement: Supplementary file 1 — Supporting Information [file ADVS-7-1903657-s001.pdf]

Copyright WILEY-VCH Verlag GmbH & Co. KGaA, 69469 Weinheim, Germany, 2020.

ADVANCED SCIENCE Open Access

Supporting Information

for *Adv. Sci.*, DOI: 10.1002/advs.201903657

Hepatocyte TMEM16A Deletion Retards NAFLD Progression by Ameliorating Hepatic Glucose Metabolic Disorder

Jia-Wei Guo, Xiu Liu, Ting-Ting Zhang, Xiao-Chun Lin, Yu Hong, Jie Yu, Qin-Yan Wu, Fei-Ran Zhang, Qian-Qian Wu, Jin-Yan Shang, Xiao-Fei Lv, Jing-Song Ou, Jia-Guo Zhou, Rui-Ping Pang, Bao-Dong Tang,* and Si-Jia Liang**

Copyright WILEY-VCH Verlag GmbH & Co. KGaA, 69469 Weinheim, Germany, 2020.

Supporting Information

Hepatocyte TMEM16A Deletion Retards NAFLD Progression by Ameliorating Hepatic Glucose Metabolic Disorder

Jia-Wei Guo, Xiu Liu, Ting-Ting Zhang, Xiao-Chun Lin, Yu Hong, Jie Yu, Qin-Yan Wu, Fei-Ran Zhang, Qian-Qian Wu, Jin-Yan Shang, Xiao-Fei Lv, Jing-Song Ou, Jia-Guo Zhou, Rui-Ping Pang, Bao-Dong Tang*, and Si-Jia Liang**

Table S1. Clinical information of all subjects included in this study

Parameter	Normal	NAFLD
All (n)	5	18
Male	4 (80.0%)	13 (72.2%)
Age (years)	35.7 ± 10.5	48.9 ± 7.6
BMI (kg/m^2)	21.4 ± 3.8	30.3 ± 3.7*
AST (IU/L)	18.6 ± 8.1	35.6 ± 6.3*
ALT (IU/L)	30.4 ± 7.7	68.5 ± 10.1*
Cholesterol (mg/dL)	104.5 ± 14.1	174.1 ± 13.5*
Triglycerides (mg/dL)	122.2 ± 9.7	181.5 ± 14.3*
Fasting blood glucose (mmol/L)	5.2 ± 1.4	7.5 ± 2.1
NAFLD score	0	4.4 ± 0.5*
Steatosis grade (0/1/2/3)	5/0/0/0	1/8/6/3
Lobular inflammation (0/1/2/3)	5/0/0/0	1/6/8/3
Ballooning (0/1/2)	5/0/0	4/8/6

$^*P < 0.05$ vs. normal. BMI, body mass index.

Table S2. Primers used for real-time PCR

Gene	Primer	Sequence
TMEM16A	Forward	5'-GTGCCACTGTGGACGAAAAC-3'
(mouse)	Reverse	5'-CAAAAAGGTGACTGGCACGG-3'
TMEM16A	Forward	5'-TGGCGTCCAAGTTCCTGACC-3'
(human)	Reverse	5'-TCGATGGCGCAGATGTTGAT-3'
CFTR	Forward	5'-TCCATTCCCAGAACCCATGC-3'
	Reverse	5'-AGGATCACTTGCCACAGAGC-3'
ClC-2	Forward	5'-AAACTCGGTTCCGACTCGAC-3'
	Reverse	5'-ACAATTCGGTAGGTGCTGCT-3'
ClC-3	Forward	5'-CATGTTGCTCTGCACCTCACT-3'
	Reverse	5'-ACACACCAGAGAGGTTTCGT-3'
SREBP-1c	Forward	5'-CACTTCTGGAGACATCGCAAAC-3'
	Reverse	5'-ATGGTAGACAACAGCCGCATC-3'
FAS	Forward	5'-CTGCGGAAACTTCAGGAAATG-3'
	Reverse	5'-GGTTCGGAATGCTATCCAGG-3'
ACCα	Forward	5'-GGCCAGTGCTATGCTGAGAT-3'
	Reverse	5'-AGGGTCAAGTGCTGCTCCA-3'
HMGCR	Forward	5'-CAGAGGCTGCAGAGCCATAA-3'
	Reverse	5'-TTCAGCAGTGCTTTCTCCGT-3'
CPT1α	Forward	5'-AGGACCCTGAGGCATCTATT-3'
	Reverse	5'-ATGACCTCCTGGCATTCTCC-3'
PPARα	Forward	5'-CTGGTCTTAACCGGCCCAAT-3'
	Reverse	5'-TGCACATAGCCAGAAGGGTG-3'
TNF-α	Forward	5'-CCTCTCATGCACCACCATCA-3'
	Reverse	5'-GCATTGCACCTCAGGGAAGA-3'
IL-1β	Forward	5'-CTTTCCCGTGGACCTTCCAG-3'
	Reverse	5'-AATGGGAACGTCACACACCA-3'
IL-6	Forward	5'-GAGACTGGGGATGTCTGTAGC-3'
	Reverse	5'-TCACCAGCATCAGTCCCAAG-3'
MCP-1	Forward	5'-TAAAAACCTGGATCGGAACCAAA-3'
	Reverse	5'-GCATTAGCTTCAGATTTACGGGT-3'
MPO	Forward	5'-TTTCTGTGCACCCCTAACCC-3'
	Reverse	5'-GGCACCATTGGAGGTCTGAA-3'
IL-10	Forward	5'-GCATGGCCCAGAAATCAAGG-3'
	Reverse	5'-AATCGATGACAGCGCCTCAG-3'
PEPCK	Forward	5'-TCAGCTGCATAACGGTCTGG-3'

	Reverse	5'-GTGGATATACTCCGGCTGGC-3'
G6Pase	Forward	5'-TCGCGCTTGGATTCTACCTG-3'
	Reverse	5'-GCCGCTCACACCATCTCTTA-3'
GLUT1	Forward	5'-CTGTTTGTTGTAGAGCGAGCTGG-3'
	Reverse	5'-AACAGCTCGGCCACAATGAAC-3'
GLUT2	Forward	5'-CCAGTTCGGCTATGACATCGG-3'
	Reverse	5'-CGAGCCACCCACCAAAGAA-3'
GLUT3	Forward	5'-TGCCCTCTGGTCCTTATGTGTG-3'
	Reverse	5'-GGCCCAGGATCAGCATTTCA-3'
GLUT4	Forward	5'-CGTCGGGTTTCCAGCAGAT-3'
	Reverse	5'-GGTGCCTTGTGGGATGGAAT-3'
VAMP1	Forward	5'-CCTCCTCCCAACATGACCAGTA-3'
	Reverse	5'-CACTACCACGATGATGGCACAG-3'
VAMP2	Forward	5'-CTGCACCTCCTCCAAACCTTAC-3'
	Reverse	5'-CCACCAGTATTTGCGCTTGAG-3'
VAMP3	Forward	5'-GGCAGTAATCGAAGACTCCAGC-3'
	Reverse	5'-GACACTGATCCCTATCGCCCA-3'
VAMP4	Forward	5'-CCACCGCTTTCAGCAACAGA-3'
	Reverse	5'-CAAGTGCAGGATGATCTGAGTGA-3'
VAMP5	Forward	5'-GTTGGAGCAGCGTTCAGACC-3'
	Reverse	5'-TCGGAAGAAAGACGACCAGC-3'
VAMP7	Forward	5'-GTTGCTCAACGTGGAGAAAGGT-3'
	Reverse	5'-CATGGCTCGAGCAAGATTTCTG-3'
VAMP8	Forward	5'-CCGAGTTAGGAACCTGCAGAGT-3'
	Reverse	5'-ACCTTCTGGGACGTTGTCTTGA-3'
GAPDH	Forward	5'-AGGAGCGAGACCCCACTAACA-3'
(mouse)	Reverse	5'-AGGGGGGCTAAGCAGTTGGT-3'
GAPDH	Forward	5'-GCCAAAAGGGTCATCATCTC-3'
(human)	Reverse	5'-TTTGGCAGGTTTTTCTAGACG-3'

SREBP-1c, sterol regulatory element-binding protein 1c; FAS, fatty acid synthase; ACCα, acetyl CoA carboxylase α; HMGCR, 3-hydroxy-3-methylglutaryl CoA reductase; TNF-α, tumor necrosis factor-α; MCP-1, monocyte chemoattractant protein 1; MPO, myeloperoxidase.

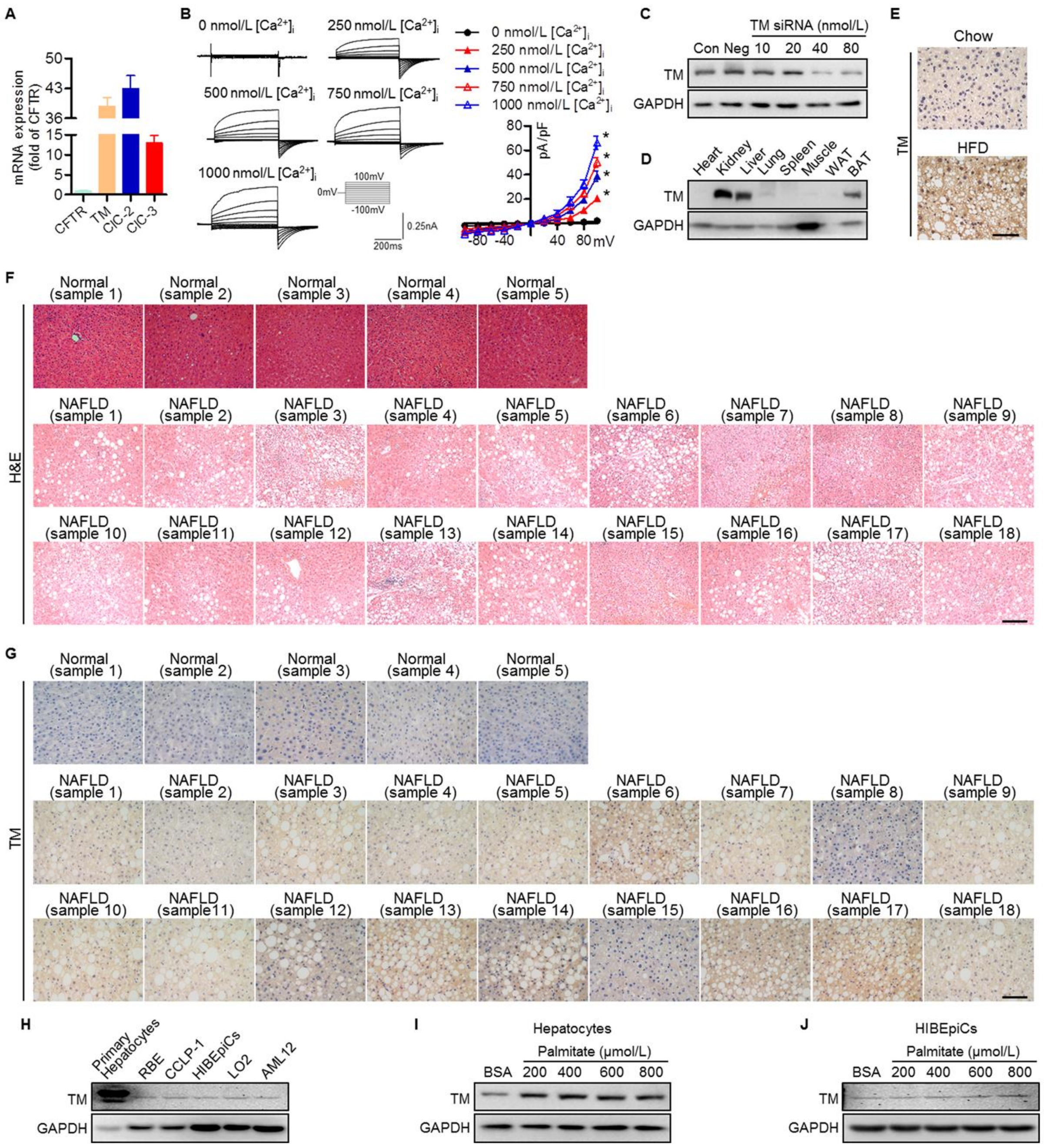

Figure S1. TMEM16A expression is increased in the fatty livers of mice and patients with NAFLD. A Abundance of CFTR, TMEM16A, ClC-2, and ClC-3 in primary hepatocytes, n = 4. **B** Representative traces of whole-cell patch-clamp recording of $I_{Cl.Ca}$ in primary hepatocytes with pipette solution buffered to the indicated $[Ca^{2+}]_i$. I-V curves of $I_{Cl.Ca}$ are shown. *$P < 0.05$ vs. 0 nmol/L $[Ca^{2+}]_i$, n = 6. **C** Western blotting of TMEM16A (TM) expression in primary hepatocytes transfected with TMEM16A siRNA or negative siRNA (Neg) for 24 h, n = 5. **D** Tissue distribution of TMEM16A, n = 4. WAT, white adipose tissue;

BAT, brown adipose tissue. **E** Immunohistochemical staining of TMEM16A in liver sections of mice fed with HFD or chow diet for 32 weeks, n = 4 per group. Representative images are shown. Scale bar, 50 µm. **F, G** Representative images of H&E (F) (scale bar, 100 µm) and TMEM16A (G) (scale bar, 50 µm) staining of liver sections from normal controls (n = 5) and patients with NAFLD (n = 18). **H** Western blot analysis of TMEM16A abundance in mouse primary hepatocytes, human hepatocyte cell line LO2, mouse hepatocyte cell line AML12, human intrahepatic biliary epithelial cells (HIBEpiCs), and cholangiocarcinoma cell lines RBE and CCLP-1, n = 4. **I, J** Primary hepatocytes (I) or HiBEpiCs (J) were treated with various concentrations of palmitate conjugated to BSA for 24 h, after which TMEM16A protein expression was determined, n = 4.

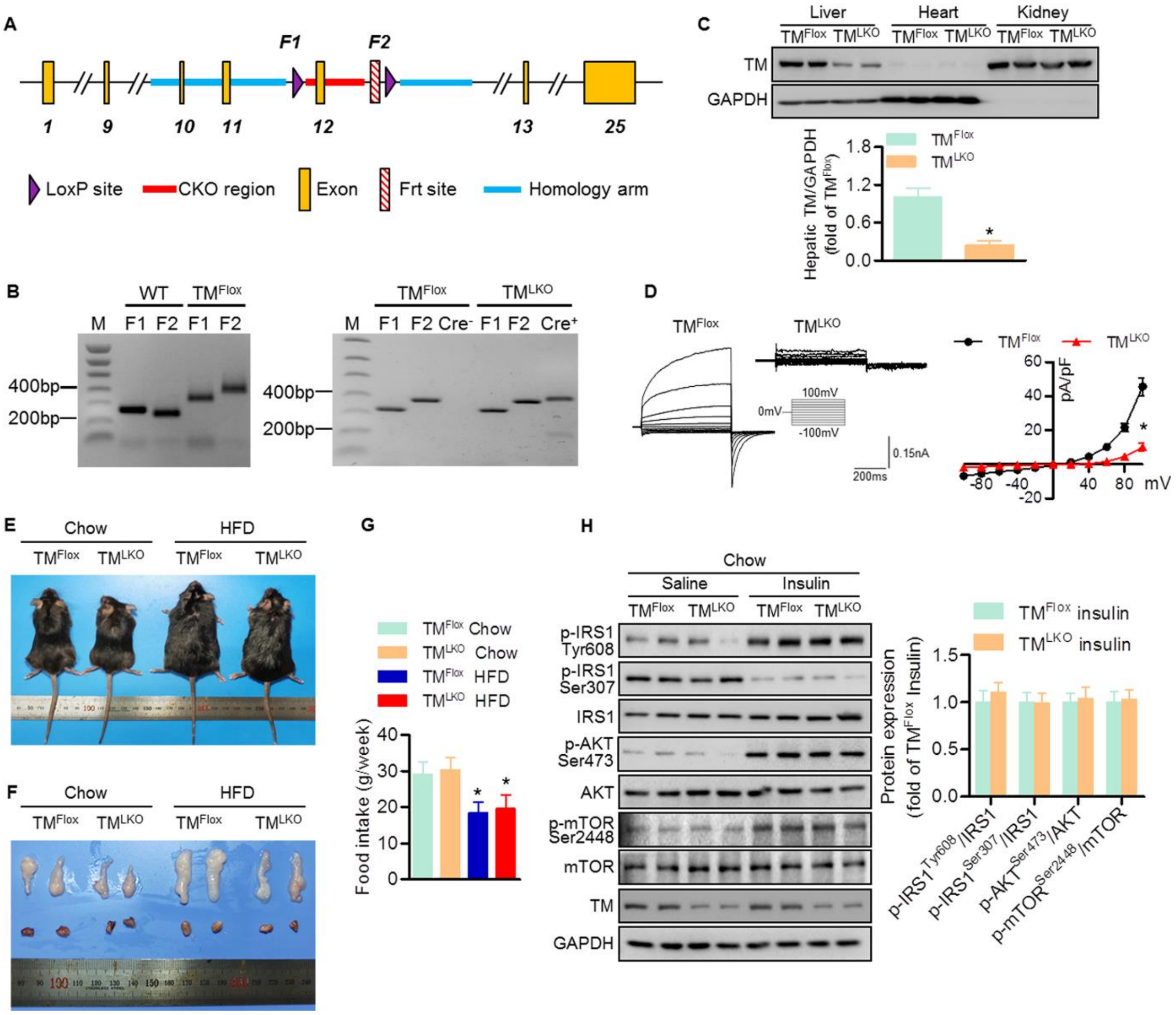

Figure S2. Hepatocyte-specific ablation inhibits the HFD-induced increase in body weight. A Genotyping strategy of TMEM16A flox (TM^{Flox}) mice. **B** Mice were identified by PCR amplifying mouse genomic DNA using specific primers for flox1 (F1), flox2 (F2), and Albumin-Cre. Mice containing flox1, flox2, and Albumin-Cre were considered TM^{LKO}. Mice containing flox1 and flox2, but not Albumin-Cre, were considered TM^{Flox}. The wild type (WT) allele produced bands of 264 and 239 bp, and the floxed allele produced bands of 325 and 379 bp.Cremice had a band of 390 bp. **C** TMEM16A abundance in the liver, heart, and kidneys of TM^{LKO} and TM^{Flox} mice. *P < 0.05 vs. TM^{Flox}, n = 4 per group. **D** $I_{Cl.Ca}$ evoked by 500 nmol/L $[Ca^{2+}]_i$ was completely inhibited in hepatocytes isolated from TM^{LKO} mice. Representative traces of $I_{Cl.Ca}$ and I-V curve of current densities are shown. *P < 0.05 vs. control, n = 4. **E, F** Representative images of body size (E) and adipose tissues (F) of TM^{Flox}

and TM^{LKO} mice fed with chow diet or HFD for 32 weeks. **G** Daily food intake during the feeding period. *$P < 0.05$ vs. TM^{Flox} chow, n = 14–18 per group. **H** Phosphorylation of IRS1 (Tyr608, Ser307), AKT (Ser473), and mTOR (Ser2448) in response to an intraperitoneal injection of saline or insulin (1.0 IU/kg for 15 min) in the liver tissues of TM^{Flox} and TM^{LKO} mice after chow diet treatment for 32 weeks, n = 5 per group.

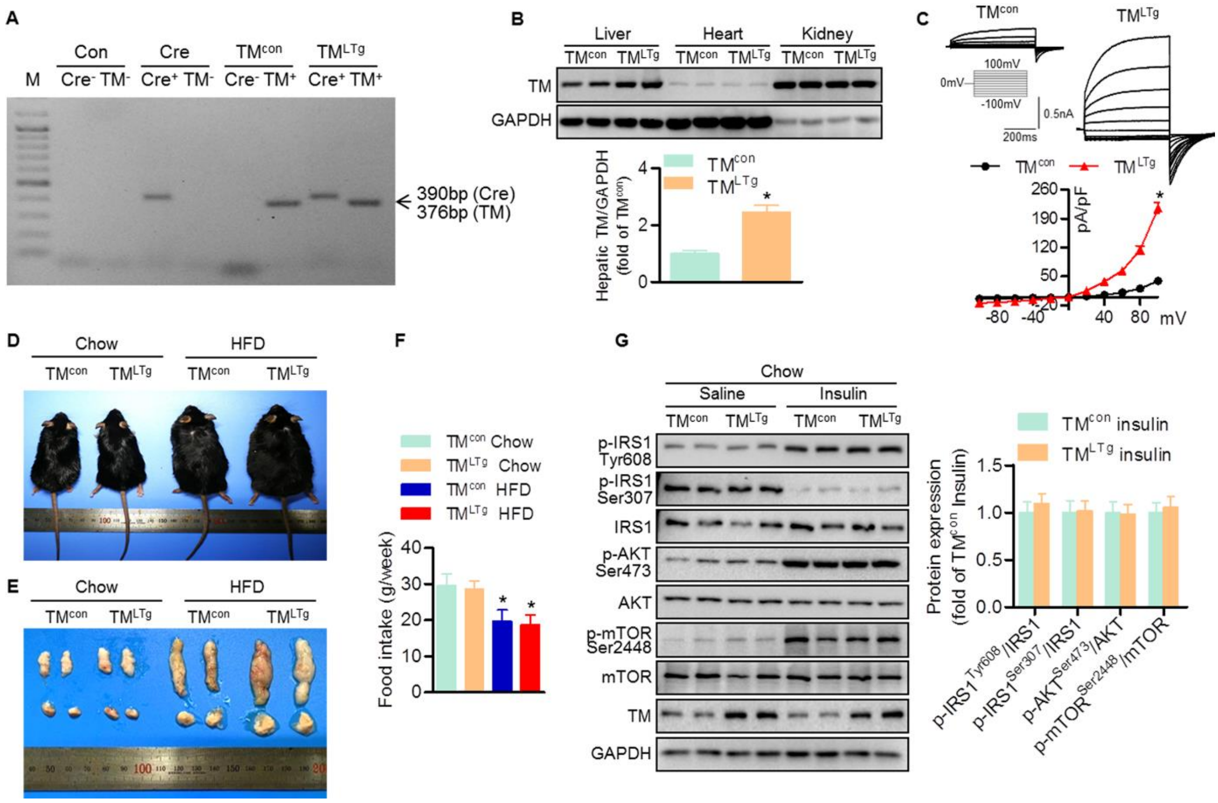

Figure S3. Hepatocyte-specific TMEM16A overexpression promotes obesity. A Identification of liver-specific TMEM16A transgenic mice. Mice were identified by PCR, amplifying mouse genomic DNA using specific primers for transgene-TMEM16A and Albumin-Cre. Mice containing transgene-TMEM16A and Albumin-Cre were considered TM^{LTg}. Mice containing transgene-TMEM16A, but not Albumin-Cre, were considered TM^{con}. The WT allele produced no band, while the TM^{con} mice produced a band of 376 bp.Cre^{+} mice had a band at 390 bp, while no band represented Cre^{-} mice.**B** TMEM16A abundance in the liver, heart, and kidneys of TM^{LTg} and their control littermates TM^{con}. *$P < 0.05$ vs. TM^{con}, n = 4 per group. **C** $I_{Cl.Ca}$ evoked by 500 nmol/L $[Ca^{2+}]_i$ was further potentiated in hepatocytes from TM^{LTg} mice compared with those from TM^{con} mice. *$P < 0.05$ vs. control, n = 4.**D**, **E** Representative images of body size (d) and adipose tissues (e) of TM^{con} and TM^{LTg} mice on the indicated diets for 32 weeks. **F** Daily food intake during the feeding period. *$P < 0.05$ vs. TM^{con} chow, n = 15–20 per group. **G** Insulin-induced IRS1-mTOR axis activation was

comparable between the liver tissues of TM^{con} and TM^{LTg} mice after 32 weeks of chow diet treatment. n = 5 per group.

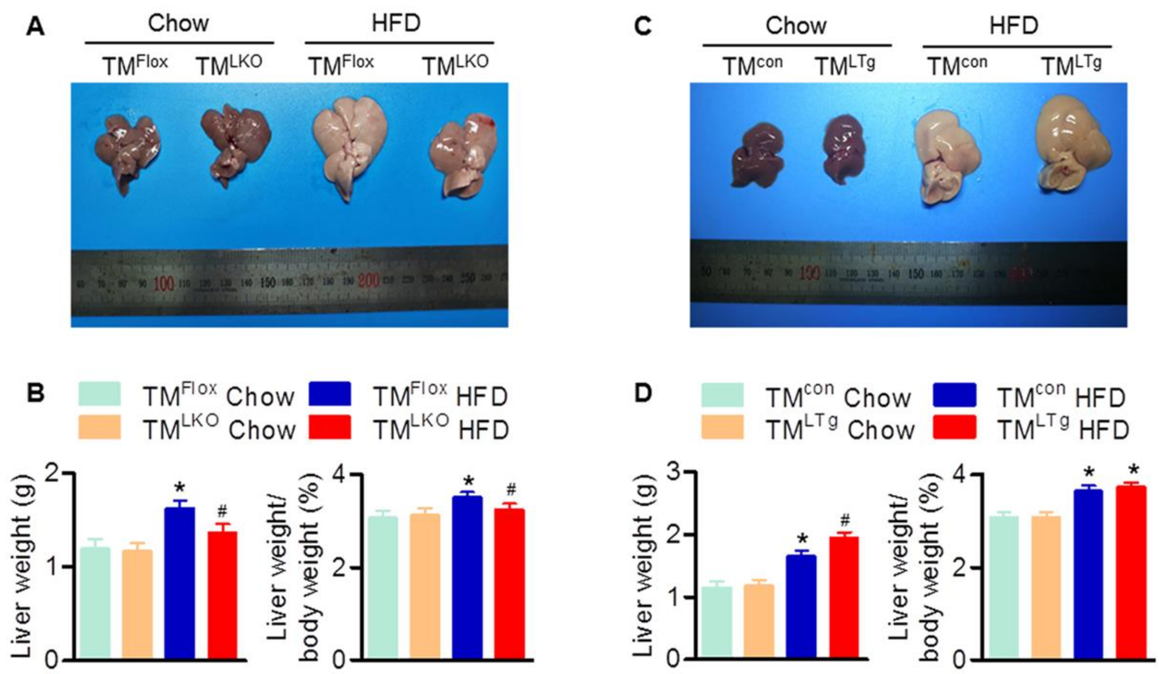

Figure S4. Hepatocyte-specific TMEM16A deletion inhibits the HFD-induced increase in liver weight. A Representative images of liver size of TM^{Flox} and TM^{LKO} mice on the indicated diets for 32 weeks. **B** HFD-induced an increase in liver weight (left) and theratio of liver weight to body weight (right) were attenuated in TM^{LKO} mice compared to those in TM^{Flox} mice. *P < 0.05 vs. TM^{Flox} chow, #P < 0.05 vs. TM^{Flox} HFD, n = 6–8 per group. **C** Representative images of liver sizes of TM^{con} and TM^{LTg} mice on the indicated diets for 32 weeks. **D** Liver-specific TMEM16A overexpression further potentiated an increase in liver weight without altering the increased liver weight to body weight ratio. *P < 0.05 vs. TM^{con} chow, #P < 0.05 vs. TM^{con} HFD, n = 10–12 per group.

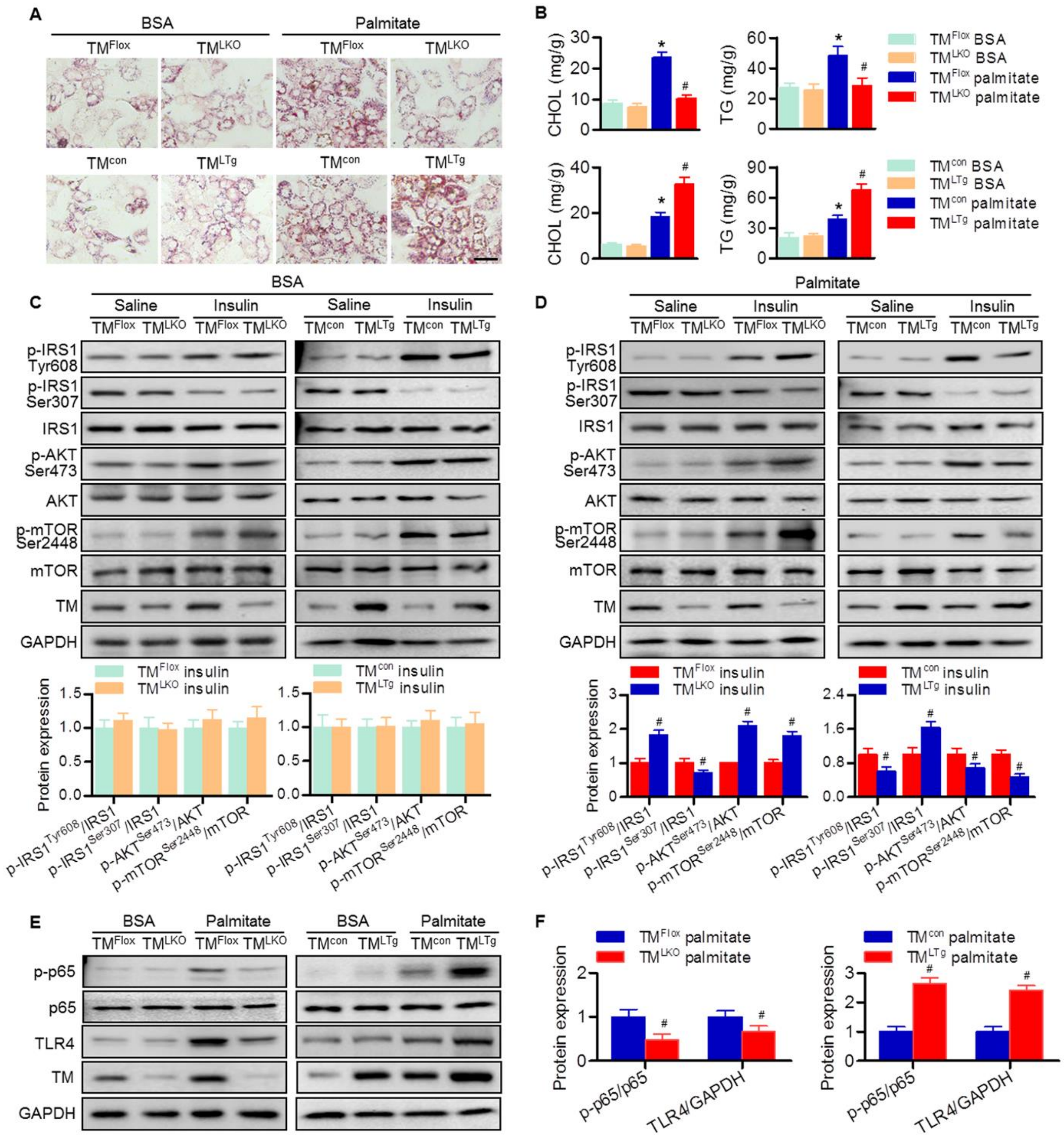

Figure S5. TMEM16A exacerbates hepatic lipid accumulation, insulin signaling dysfunction, and inflammatory response in hepatocytes. A, **B** Lipid deposition (A), cholesterol content (CHOL), and triglyceride levels (TG) (B) in primary hepatocytes from TM^{LKO} mice, TM^{LTg} mice, and their control littermates treated with BSA or palmitate (200 μmol/L) for 24 h. Scale bar, 20 μm. *P < 0.05 vs. TM^{Flox} BSA or TM^{con} BSA, #P < 0.05 vs. TM^{Flox}palmitate or TM^{con}palmitate, n = 6.**C**, **D** Hepatocytes from TM^{LKO} mice, TM^{LTg} mice, and their control littermates were incubated with BSA (C) or palmitate (D) for 24 h, followed by insulin (100 nmol/L) stimulation for 30 min. Phosphorylation of IRS1 (Tyr608, Ser307),

AKT (Ser473), and mTOR (Ser2448) was determined by western blotting. #$P < 0.05$ vs. TM^{Flox} insulin or TM^{con} insulin, n = 6. **E**, **F** p65 phosphorylation and TLR4 protein expression inhepatocytes treated as described. #$P < 0.05$ vs. TM^{Flox}palmitate or TM^{con}palmitate, n = 6.

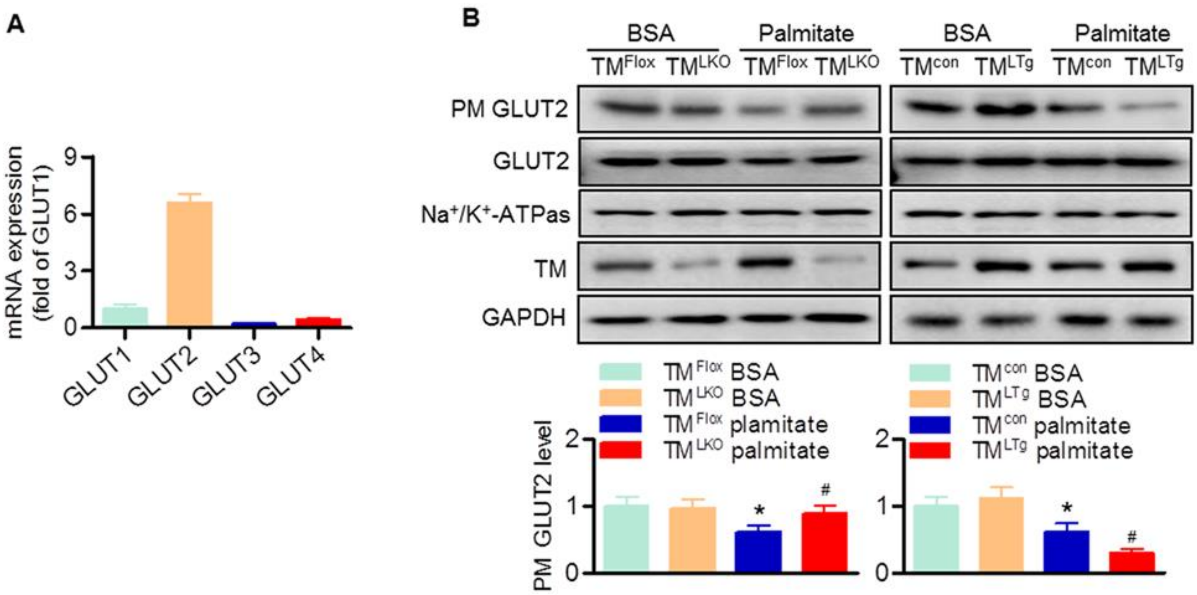

Figure S6. TMEM16A deficiency ameliorates the palmitate-induced inhibition of GLUT2 translocation in hepatocytes. A Abundance of GLUT1, GLUT2, GLUT3, and GLUT4 in primary hepatocytes, n = 5. **B** Hepatocytes from the four mouse groups were treated with BSA or palmitate for 24 h followed by incubation in culture medium without glucose for 6 h. Afterward, the medium was replaced with high glucose DMEM and incubated for 2 h. Plasma membrane (PM) and total GLUT2 content were measured. **P* < 0.05 vs. TM^{Flox} BSA or TM^{con} BSA, #*P* < 0.05 vs. TM^{Flox}palmitate or TM^{con}palmitate, n = 5.

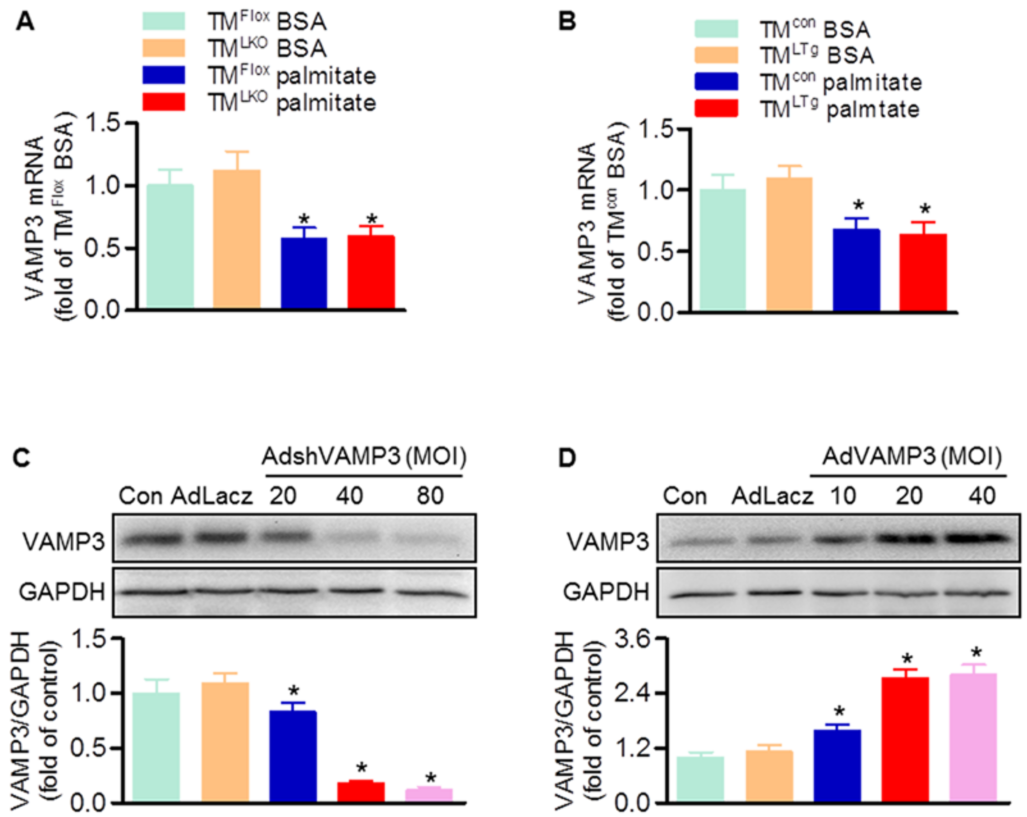

Figure S7. TMEM16A knockout or overexpression does not alter the palmitate-induced decrease in VAMP3 mRNA level. A, **B** mRNA expression of VAMP3 in hepatocytes from TM^{LKO} mice (A), TM^{LTg} mice (B), and their control littermates treated with BSA or palmitate (200 µmol/L) for 24 h. **P* < 0.05 vs. TM^{Flox} BSA or TM^{con} BSA, n = 4. **C**, **D** Hepatocytes were infected with different multiplicities of infection (MOI) of AdshVAMP3 (C) or AdVAMP3 (D) for 24 h. VAMP3 protein expression was examined. **P* < 0.05 vs. control, n= 4.
